# Late Embryogenesis Abundant Proteins Contribute to the Resistance of Toxoplasma gondii Oocysts against Environmental Stresses

**DOI:** 10.1128/mbio.02868-22

**Published:** 2023-02-21

**Authors:** David Arranz-Solís, David Warschkau, Benedikt T. Fabian, Frank Seeber, Jeroen P. J. Saeij

**Affiliations:** a Pathology, Microbiology and Immunology Department, School of Veterinary Medicine, University of California Davis, Davis, California, USA; b Mycotic and Parasitic Agents and Mycobacteria (FG16), Robert Koch Institute, Berlin, Germany; Albert Einstein College of Medicine

**Keywords:** *Toxoplasma gondii*, oocysts, sporozoite, environmental resistance, late embryogenesis abundant proteins, intrinsically disordered proteins

## Abstract

Toxoplasma gondii oocysts, which are shed in large quantities in the feces from infected felines, are very stable in the environment, resistant to most inactivation procedures, and highly infectious. The oocyst wall provides an important physical barrier for sporozoites contained inside oocysts, protecting them from many chemical and physical stressors, including most inactivation procedures. Furthermore, sporozoites can withstand large temperature changes, even freeze-thawing, as well as desiccation, high salinity, and other environmental insults; however, the genetic basis for this environmental resistance is unknown. Here, we show that a cluster of four genes encoding Late Embryogenesis Abundant (LEA)-related proteins are required to provide *Toxoplasma* sporozoites resistance to environmental stresses. *Toxoplasma* LEA-like genes (*TgLEAs*) exhibit the characteristic features of intrinsically disordered proteins, explaining some of their properties. Our *in vitro* biochemical experiments using recombinant TgLEA proteins show that they have cryoprotective effects on the oocyst-resident lactate dehydrogenase enzyme and that induced expression in E. coli of two of them leads to better survival after cold stress. Oocysts from a strain in which the four *LEA* genes were knocked out *en bloc* were significantly more susceptible to high salinity, freezing, and desiccation compared to wild-type oocysts. We discuss the evolutionary acquisition of *LEA*-like genes in *Toxoplasma* and other oocyst-producing apicomplexan parasites of the *Sarcocystidae* family and discuss how this has likely contributed to the ability of sporozoites within oocysts to survive outside the host for extended periods. Collectively, our data provide a first molecular detailed view on a mechanism that contributes to the remarkable resilience of oocysts against environmental stresses.

## INTRODUCTION

Toxoplasma gondii is a protozoan parasite that can infect virtually all birds and mammals, including humans. Although most infected healthy individuals have no symptoms, it can cause severe disease in immunocompromised or congenitally infected patients, and neonatal mortality/miscarriages if a pregnant woman gets infected for the first time (1, 2). In addition, toxoplasmosis causes important economic losses in the livestock sector related to reproductive failure, mainly in sheep and goats (3). Infection can occur after ingestion of tissue cysts present in meat or viscera from infected animals or of water or food contaminated with oocysts shed in the feces of an infected feline (definitive host) (4). Once in the environment, and under favorable conditions of humidity, aeration, and temperature, sporozoites develop within oocysts. The mature (or sporulated) *Toxoplasma* oocyst has in its final form two sporocysts containing four sporozoites each. Besides being highly stable in the environment and extremely resistant to inactivation procedures, oocysts are exceptionally infectious to intermediate hosts, as even a single oocyst can elicit the infection (5, 6).

Waterborne toxoplasmosis due to ingestion of oocysts has been associated with large outbreaks in animals and humans, often with severe symptoms (7, 8). In addition, toxoplasmosis cases have been reported in strictly vegetarian human subpopulations (9). Clinical toxoplasmosis has also been reported in marine mammals and species from coastal habitats, such as seals and sea otters (10, 11). These infections are usually caused by oocysts that are mobilized from fecal depositions of felids to rivers and watersheds that drain into coastal waters, especially via runoffs after heavy rainfalls (12–14). In this sense, *Toxoplasma* oocysts have been shown to survive for several months in seawater, suggesting salt resistance and explaining, in turn, the detection of oocysts in marine invertebrates such as mussels and oysters (15, 16), which can increase the risk of *Toxoplasma* infection for people and marine wildlife (17).

The bilayered oocyst and sporocyst walls provide an important protective barrier and likely shield the sporozoites from chemical and physical stressors (18). Indeed, the oocyst wall outer coating contains acid-fast lipids that are thought to constitute a permeability barrier to water-soluble molecules, such as disinfectants and detergents, while these lipids are sensitive to polar molecules and organic solvents, explaining the well-described loss of the outer layer after bleach treatments (18, 19). On the other hand, the inner layer contains cysteine-rich proteins, also named oocyst wall proteins or OWPs (20, 21); tyrosine-rich proteins, which are responsible for the characteristic UV light-induced auto-fluorescence of oocyst and sporocyst walls typical of coccidian oocysts (18, 22, 23); and β-1,3-glucan fibrils and proteins, which are thought to provide stability to the oocyst wall (18, 24). The cross-linked network of tyrosine-rich proteins has been shown to be involved in the stabilization of various extracellular matrices, such as insect and nematode cuticles and yeast cell walls, suggesting a likely implication in the hardening of the oocyst wall (25, 26).

Table 1 summarizes the most relevant studies describing the resistance of *Toxoplasma* oocysts against different temperatures and chemical compounds. For example, *Toxoplasma* oocysts can withstand freezing conditions for weeks and temperatures between 40 and 45°C for several days (27, 28). Similarly, most disinfectants are not able to inactivate oocysts, including bleach, ethanol, or formalin, and only ammonia compounds seem to be efficient in inactivating oocysts (27, 29, 30). Of note, it seems that unsporulated oocysts are much more sensitive and do not resist these treatments. For instance, it has been shown that unsporulated oocysts do no longer sporulate if simply kept at 37°C for as short as 24 h (31, 32). Similarly, many disinfectants that failed to inactivate sporulated oocysts can prevent oocysts from sporulating and thus from becoming infectious (30, 31, 33). Hence, oocysts must be mature, or sporulated, to acquire their characteristic resistance to environmental insults. It is then plausible that the oocyst wall alone, which is already present in unsporulated oocysts, cannot explain sporozoite protection against desiccation, temperature changes, or generally staying viable for prolonged times outside a host.

**TABLE 1:** Resistance treatments reported in the literature for *Toxoplasma* sporulated oocysts^*a*^

Treatment	Strain (age oocysts)	No. of oocysts tested	Complete inactivation in	Comments	Reference
−80°C	NO-1 or Fukaya (UK)	UK	Less than 2 wks (no earlier times tested)		32
−21°C	M-7741 (fresh)	5,000	More than 4 wks (no longer times tested)		111
−20°C	NO-1 or Fukaya (UK)	UK	5−6 wks		32
−20°C	UK	UK	3 wks		112
−10°C	VEG (UK)	100,000	More than 3–4 mo	Water bath with fluctuation of temp	28
−5°C	NO-1 or Fukaya (UK)	UK	Estimated to be more than, but close to, 4 mo		32
0°C	VEG (UK)	100,000	More than 13 mo		28
4°C	VEG (UK)	100,000	More than 54 mo		28
30°C	VEG (UK)	100,000	More than 3–4 mo		28
35°C	VEG (UK)	100,000	2 mo		28
37°C	Beverley (UK)	UK	10 mo		151
40°C	VEG (UK)	100,000	1 mo		28
45°C	VEG (UK)	100,000	2 days		28
50°C	M-7741 (fresh)	Fecal suspension (400,000?)	More than 30 mins (no longer times tested)		27
50°C	VEG (UK)	100,000	2 h		28
50°C	O-1 and O-3 (<3 mo old)	2,500	30 mins	Contamination with *Isospora bigemina*?	152
52°C	VEG (UK)	100,000	More than 5 mins		28
55°C	M-7741 (fresh)	Fecal suspension (400,000?)	30 minutes or less (no shorter times tested)		27
55°C	VEG (UK)	100,000	2 minutes		28
55°C	O-1 and O-3 (<3 mo old)	2,500	15 minutes or less (no shorter times tested)	Contamination with *Isospora bigemina*?	152
58°C	UK	UK	15 minutes or less (no shorter times tested)		112
60°C	VEG (UK)	100,000	1 min		28
60°C	O-1 and O-3 (<3 mo old)	2,500	10−15 mins	Contamination with *Isospora bigemina*?	152
60°C (radio frequency)	type II SO (UK)	10,000-100,000 (fecal suspension)	Inconsistent results depending on time and speed	Radio-Frequency thermal treatments in water (different incubation times to reach final temp)	153
70°C	O-1 and O-3 (<3 mo old)	2,500	1−2 mins	Contamination with *Isospora bigemina*?	152
Acidified ethanol (5% acetic acid, 95% EtOH)	M-7741 (fresh)	Fecal suspension (400,000?)	Less than 24 h but more than 1 h		27
Acidified ethanol (5% acetic acid, 95% EtOH)	VEG (<6 mo old)	5,000,000	More than 24 h (no tested longer)	Room temp, tested by RT-PCR and plaque assay	110
Ammonia water (10%)	O-1 and O-3 (fresh)	2,500	30 mins or less (no shorter times tested)	Contamination with *Isospora bigemina*?	30
Ammonium hydroxide (10%)	M-7741 (fresh)	Fecal suspension (400,000?)	5 mins or less (no shorter times tested)		27
Ammonium hydroxide (28%)	M-7741 (fresh)	UK (unpurified oocysts in feces)	10 mins		29
Ammonium hydroxide (5%)	M-7741 (fresh)	Fecal suspension (400,000?)	Between 10 and 30 mins		27
Ammonium sulfide	O-1 and O-3 (fresh)	2,500	1 h	Contamination with *Isospora bigemina*?	30
Bleach (undiluted Purex, 6% Sodium hypochlorite)	M-7741 (fresh)	Fecal suspension (400,000?)	More than 24 h (no longer times tested)		27
Bleach (~5.25% NaOCl)	type II SO (UK)	100,000	More than 24 h (no longer times tested)		114
Bleach (10% Clorox, ~0.6% NaOCl, acidified)	VEG (<6 mo old)	1,000−100,000	More than 24 h (no tested longer)	Room temp, tested by RT-PCR and plaque assay	110
Bleach (10% Clorox, ~0.6% NaOCl, acidified)	VEG (<6 mo old)	1−100	24 h or less (no shorter times tested)	Room temp, tested by RT-PCR and plaque assay	110
Drying (21–23°C)	UK	UK	More than 7 wks (no longer times tested)	Uncovered strips of paper at RT	112
Drying (RT)	M-7741 (fresh)	1,000	24 h or less (no shorter times tested)	Oocysts allowed to dry for 24 h and resuspended	27
Drying (RT, 37% humidity)	M-7741 (fresh)	100,000 (estimation)	3 days		29
EtOH (70%)	O-1 and O-3 (fresh)	2,500	More than 48 h (no longer times tested)	Contamination with *Isospora bigemina*?	30
EtOH (95%)	UK	UK	More than 24 h (no longer times tested)		112
EtOH (95%, acidified)	VEG (<6 mo old)	10−100,000	More than 24 h (no tested longer)	Room temp, tested by RT-PCR and plaque assay	110
EtOH (99%)	O-1 and O-3 (fresh)	2,500	24 h	Contamination with *Isospora bigemina*?	30
Formalin (10%)	M-7741 (fresh)	Fecal suspension (400,000?)	Less than 24 h (no times between 6 and 24 h)		27
Formalin (10%)	O-1 and O-3 (fresh)	2,500	Less than 4 days	Contamination with *Isospora bigemina*?	30
Formalin (10%)	UK	UK	More than 24 h (no longer times tested)		112
Formalin (10%)	VEG (<6 mo old)	1−100,000	24 h or less (no shorter times tested)	Room temp, tested by RT-PCR and plaque assay	110
Hibisept (chlorhexidine in 70% EtOH)	UK	UK	More than 24 h (no longer times tested)		112
Household ammonia	M-7741 (fresh)	UK (unpurified oocysts in feces)	3 h		29
Izosan-G (0.02–0.04%)	UK	UK	More than 24 h (no longer times tested)		112
Lomasept (5%)	O-1 and O-3 (fresh)	2,500	3 h	Contamination with *Isospora bigemina*?	30
MetOH (100%)	O-1 and O-3 (fresh)	2,500	12 h	Contamination with *Isospora bigemina*?	30
MetOH (100%)	UK	UK	More than 24 h (no longer times tested)		112
NaCl (15 g/L)−artificial sea water at 4°C	Chicken isolate (fresh)	500,000	More than 24 mo (no longer times tested)	The same conditions at RT killed the oocysts	116
NaCl (32 g/L)−artificial sea water	Chicken isolate (fresh)	10,000	More than 6 mo (no longer times tested)	Sporulation also unaffected	33
Neo-Kurehasol (1%)	O-1 and O-3 (fresh)	2,500	48 h	Contamination with *Isospora bigemina*?	30
Nitric acid-sulfuric acid (1:1)	M-7741 (fresh)	Fecal suspension (400,000?)	24 h (no shorter times tested)		27
*n*-propyl, *n*-butyl or Isoamyl alcohols	O-1 and O-3 (fresh)	2,500	More than 48 h (no longer times tested)	Contamination with *Isospora bigemina*?	30
Ozone (6 mg/L)	type II SO (UK)	10,000	More than 12 mins (no longer times tested)		114
Ozone (9.4 mg min/L)	VEG (6 mo old)	250,000	More than 20 mins (no effect)	Bioassay in mice and *in vitro* assay	154
Peracetic acid (5%)	O-1 and O-3 (fresh)	2,500	48 h	Contamination with *Isospora bigemina*?	30
Sulfuric acid sodium dichromate (63–7%)	M-7741 (fresh)	Fecal suspension (400,000?)	Less than 24 h but more than 30 minutes		27
Tincture of iodine (7% iodine, 5% potassium iodide)	M-7741 (fresh)	UK (unpurified oocysts in feces)	30 mins		29
Tincture of iodine	O-1 and O-3 (fresh)	2,500	More than 48 h (no tested longer)	Contamination with *Isospora bigemina*?	30
UV (continuous)	type II SO (UK)	100,000	40 mJ/cm^2^		155
UV (pulsed)	type II SO (UK)	100,000	750 mJ/cm^2^		155
UV (pulsed)	type II SO (UK)	10,000	106 mJ/cm^2^		155
UV (pulsed)	VEG (<3 mo old)	5,000	10 mJ/cm^2^	Viability test by mouse bioassay, RT-qPCR, and plaque assay	156
UV continuous (40 mJ/cm2)	VEG (6 mo old)	5−50,000	4-log reduction	Bioassay in mice and plaque assay	154
Virkon-S (2%)	UK	UK	More than 24 h (no longer times tested)		112
Wescodyne (1%)	VEG (<6 mo old)	1−100,000	More than 24 h (no longer times tested)	Room temp, tested by RT-PCR and plaque assay	110

a The most relevant temperature-based and chemical/physical-based (highlighted in gray) treatments described in the literature are summarized. Oocyst viability was performed by mouse bioassay unless otherwise stated. UK, unknown (not described or omitted); SO, sea otter; TOP, *Toxoplasma* oocyst plaque assay; wks, weeks; mo: months; min, minutes; temp, temperature. The sentence “contamination with *Isospora bigemina*?” is included to warn readers that the oocysts used for this experiment were obtained from cats that had spontaneously been infected with *Isospora bigemina* (30). Therefore it is possible that this potential mixture of *Toxoplasma* and Isospora oocysts may have affected the recorded results in 30 and 152.

Late Embryogenesis Abundant (LEA) proteins were first described 4 decades ago in cotton seeds (*Gossypium hirsutum*), accumulating at high levels during the later stages of embryo development to protect the seeds from damages caused by abiotic stresses (34, 35). They were subsequently found in other plants, not only in their seeds, but also in vegetative tissues following environmental abiotic stresses, such as cold, drought, or high salinity (36, 37). Moreover, LEA homologues have also been described in other organisms, such as bacteria, helminths, and invertebrates (38), with a commonly ascribed role in the resistance to environmental stresses (39). These proteins exhibit peculiar biochemical properties, such as a biased amino acid composition that leads to high hydrophilicity, heat stability and the presence of intrinsically disordered regions (IDRs) (39–42). This allows LEA proteins to have antiaggregation, protein stabilization, as well as molecular chaperone-like activities (39, 41, 43, 44). It translates into a redirection of water molecules, binding of salt ions, elimination of active oxygen free radicals, prevention of the cell structure collapse, and repair of misassembled proteins, among others (45, 46).

In the present work we investigated the role of a cluster of four *Toxoplasma* LEA-like genes (47, 48) on oocyst resistance against environmental stresses. These gene products show many features of intrinsically disordered proteins (IDPs) as assessed by bioinformatic and biochemical means, and the individual overexpression of these four TgLEA proteins in E. coli resulted in some protection from temperature stress by two proteins of this cluster, suggesting they are genuine LEAs. We also show that sporulated oocysts from a strain lacking this entire cluster are significantly more susceptible to temperature, humidity, and salinity changes. To our knowledge, this is the first functional description of LEA-like proteins in protozoans and their role in protecting free life cycle stages from environmental stressors.

## RESULTS

### LEA genes are highly upregulated in sporozoites and might be related to the resistance of oocysts to environmental stresses.

We used transcriptomic and proteomic data from studies of different *Toxoplasma* developmental stages, which are available on ToxoDB, to identify potential genes involved in the resistance of sporulated oocysts (49). We noted that among the genes that are the highest expressed specifically in sporulated oocysts, there are four that code for putative LEA domain-containing proteins, which are part of a gene cluster on Chromosome XII of the *Toxoplasma* genome (TGME49_276850, 276860, 276870, and 276880), here called TgLEA850, TgLEA860, TgLEA870 and TgLEA880, or collectively TgLEA8x0. Inspection of the four translated genes of all 64 sequenced *Toxoplasma* haplotypes indicates very little protein sequence divergence (Fig. S1). Like LEA proteins from other species, the putative *Toxoplasma* LEA proteins have intrinsically disordered regions, a high proportion of polar amino acids (aa) and high hydrophilicity, among other properties (see next paragraph). Since not many LEA-designated proteins have been functionally characterized outside the plant system, a general problem with this annotation is that due to the highly biased aa composition (50), a relatively low sequence homology does not automatically indicate their involvement in stress response. Extensive *in silico* and experimental analyses of the TgLEA8x0 genes and proteins were therefore initiated to verify their assignments.

10.1128/mbio.02868-22.1 FIG S1 Protein alignments of the sequence of the 64 haplotypes of *Toxoplasma*, illustrated as sequence logo. Only shaded amino acids (cyan) are different. Note that sequencing errors in some genome sequences might be responsible for differences in some cases. Download FIG S1, TIF file, 2.7 MB. Copyright © 2023 Arranz-Solís et al. 2023 Arranz-Solís et al. https://creativecommons.org/licenses/by/4.0/ This content is distributed under the terms of the Creative Commons Attribution 4.0 International license.

### TgLEA8x0 are predicted LEA class 6 IDPs.

**FIG 1:**
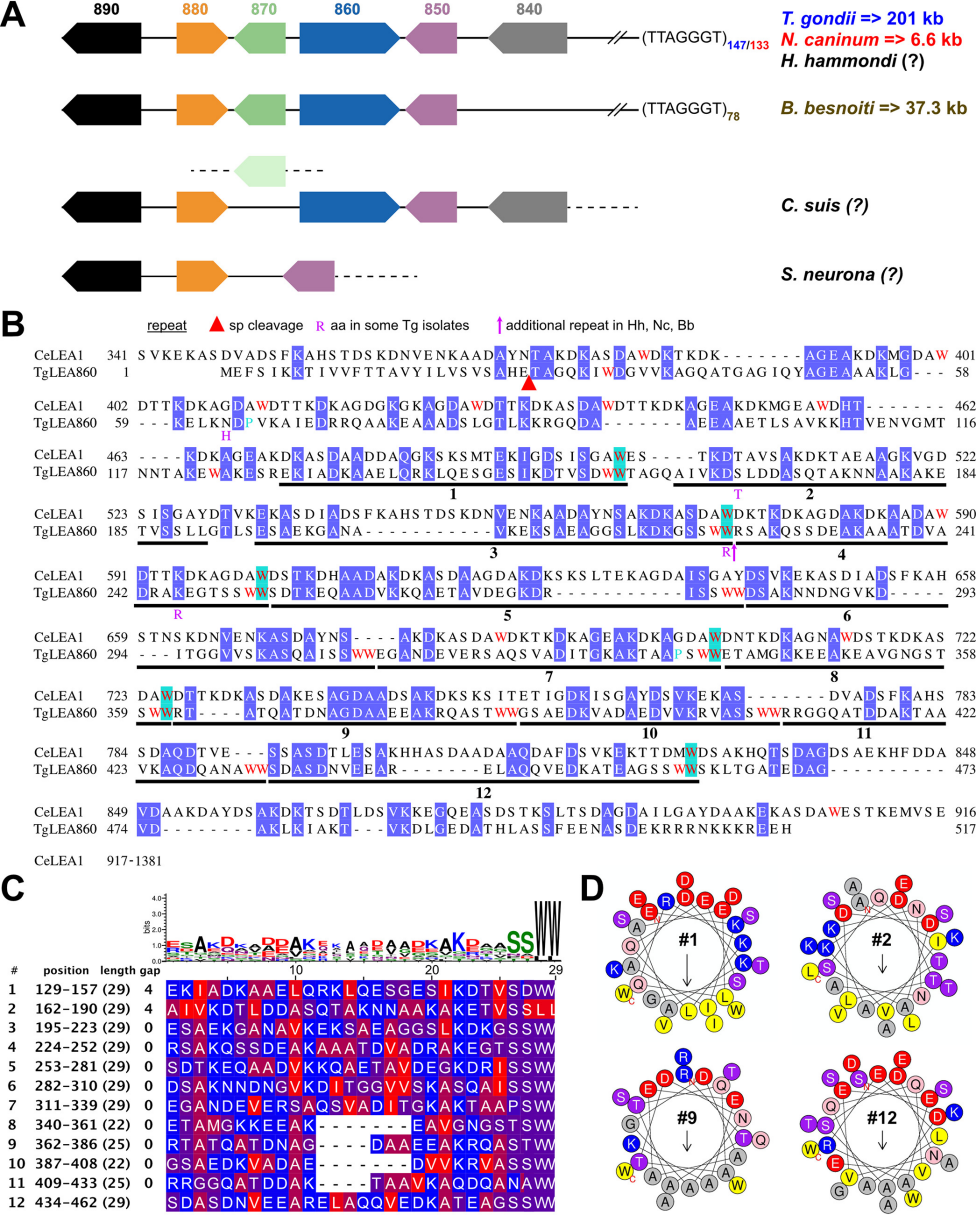
Comparisons of TgLEA8x0 synteny and primary sequence features of TgLEA860 with CeLEA1. (A) Schematic synteny of *TgLEA850*, -*860*, -*870* and -*880* genes, and the adjacent TGME49_276840 and TGME49_276890 genes as references, with other members of the *Sarcocystidae* family. TTAGGGT denotes the telomere sequence; and the subscript numbers their frequency. => gives the distance to the first telomere sequence in kilobases, whereas the question mark (?) indicates that this information was not available. The *LEA870* sequence of *C. suis* (light green symbol) is on another contig. (B) Sequence alignment of TgLEA860 with LEA-1 isoform l from C. elegans (NP_001256160, encompassing aa 348 to 903). Identical residues are shaded in blue; tryptophans (W) are colored in red, with identical positions highlighted in cyan. Further features are explained in the graph. sp: signal peptide. (C) Alignment of TgLEA860 repeats. The JalView hydrophobicity scheme color code was used (blue, most hydrophilic; red, most hydrophobic). Sequence logo on top represents the individual frequency of the residues. (D) Helical wheel representation of repeats 1, 2, 9 and 12 as examples of the amphipathic nature of the repeats. Amino acid color code: blue indicates positive; red, negative; pink, polar and G, L, Y; and gray and yellow, nonpolar and hydrophobic residues, respectively.

When strict criteria like significant sequence identity (above 30%) (Fig. S2A) and clear synteny (Fig. 1A; see also “LEA8x0 genes from *Sarcocystidae* are subtelomeric and putative *de novo* genes”) were applied, the cluster of the four LEAs is confined to the *Sarcocystidae* family, and the divergence in sequence and synteny within the cluster is consistent with the organisms’ phylogenetic positions. Primary sequence homologies between LEAs are limited (Fig. S3A–D), and BLAST and DELTA-BLAST searches indicated some homology with proteins annotated as LEA proteins from plants for TgLEA860 and TgLEA880, but not for the other two proteins (see next paragraph). BLAST searches on LEApDB (50) resulted in low-to-medium homologies (<30%) to assigned LEAs from other organisms, all of which belong to class 6. However, only TgLEA860 shows a class 6 motif, as defined by Jaspard et al. (50) (Table S1).

10.1128/mbio.02868-22.2 FIG S2 Sequence comparison of the four LEA proteins across species and their identifiers. (A) Sequence identities between the four LEA proteins from the *Sarcocystidae* species, calculated for ungapped parts of the alignments. (B) Compilation of the gene identifiers for all species in the ToxoDB and OrthoMCL databases. Download FIG S2, TIF file, 1.1 MB. Copyright © 2023 Arranz-Solís et al. 2023 Arranz-Solís et al. https://creativecommons.org/licenses/by/4.0/ This content is distributed under the terms of the Creative Commons Attribution 4.0 International license.

10.1128/mbio.02868-22.3 FIG S3 Comparison of protein sequence features of the four LEA genes across *Sarcocystidae*. (A to D) Sequence identities between the orthologous LEA proteins from the respective species. Identical residues are shaded in blue; tryptophans (W) in magenta; the “missing (B) or extra (D) repeats” are shaded in cyan. Repeats are indicated by numbered lines. In panels A and C, the % identity and similarity, when aligned to each other, are given. For the repeats of TgLEA860 see Fig. 1B, and for gene accession numbers see Fig. S4B. (D) The LEA880-like protein sequence of the *Eimeriidae* is shown below the dotted line (for gene accession numbers see Fig. S6A) and aligned with those of the other LEA880 sequences. Download FIG S3, TIF file, 4.8 MB. Copyright © 2023 Arranz-Solís et al. 2023 Arranz-Solís et al. https://creativecommons.org/licenses/by/4.0/ This content is distributed under the terms of the Creative Commons Attribution 4.0 International license.

10.1128/mbio.02868-22.8 TABLE S1 Bioinformatic and biochemical data of TgLEA8x0. Download Table S1, DOCX file, 0.02 MB. Copyright © 2023 Arranz-Solís et al. 2023 Arranz-Solís et al. https://creativecommons.org/licenses/by/4.0/ This content is distributed under the terms of the Creative Commons Attribution 4.0 International license.

TgLEA860, which is much larger than the other three proteins, shows homology to other proteins. One is Synechococcus elongatus protein WP_011378017, which has 20.5% identity over a length of 492 aa and shows one LEA class 6 motif, but at a different position. Neither comparison of disorder predictions nor that of the sequence of the 12 direct repeats of 22 aa show resemblance to TgLEA860 (data not shown).

A LEA protein from C. elegans, CeLEA1 isoform l (NP_001256160) (51–53) is 27.5% identical and 40.2% similar over the entire mature sequence of TgLEA860 (Fig. 1B) (note that CeLEA1 is considerably longer at both the N- and C-terminus). The parasite protein has a predicted signal peptide, implying that it could be exported and thus become part of the sporocyst wall. Moreover, it is extremely tryptophan-rich (4.6% versus 1% mean of all *Toxoplasma* proteins; see Table S2), with 24 of the 26 W residues appearing as doublets (WW). Twelve imperfect, direct repeats of 22 to 29 aa can be defined in TgLEA860 (Fig. 1C), where WW demarcates the C-terminal end of a repeat. This is better demonstrated by inspecting a multiple alignment of the homologous LEA860 sequences from Hammondia hammondi, Neospora caninum, and Besnoitia besnoiti (Fig. S3B), where it is evident that these three species have exactly one additional repeat, inserted between repeats no. 3 and no. 4 of *Toxoplasma*. All repeats are predicted to constitute amphipathic helices (Fig. 1D), which in general can interact with lipids or proteins via their hydrophobic interfaces (54). Repetitive structures are long known in LEA proteins; indeed, an 11-mer (TAQAAKEKAXE [55]) was initially defined in plants, and a different one was found in CeLEA-1 (DKASDAWDSAK [53]). Inspection of the TgLEA860 repeats revealed some resemblance to these 11-mer sequences when slid along the 22- to 29-mers but excluding the C-terminal SSWW quadruplet. They thus constitute a new repeat motif.

10.1128/mbio.02868-22.9 TABLE S2 Predicted consensus phosphorylation sites. Download Table S2, DOCX file, 0.01 MB. Copyright © 2023 Arranz-Solís et al. 2023 Arranz-Solís et al. https://creativecommons.org/licenses/by/4.0/ This content is distributed under the terms of the Creative Commons Attribution 4.0 International license.

For TgLEA880, six direct repeats of 22 aa each, spanning the entire length of the protein, could be identified (Fig. S3D and S4A), five of which are predicted to be amphipathic helices (Fig. S4B). Again, inspection of the multiple sequence alignment of the different *Sarcocystidae* LEAs corroborates the correct assignment of the repeats since the *C. suis* homolog possesses an additional 22-aa repeat (Fig. S3D). Interestingly, although of overall low sequence identity (<30%), some resemblance to LEA880 could be found for a gene in the *Eimeriidae* family (Fig. S2A and S3D), for which also six direct repeats (seven in *Eimeria mitis*) of 22-aa length and with some predicted amphipathic properties can be assigned to (Fig. S4A, C). Whether these LEA880-like genes can be regarded as functional orthologs needs to be examined, but they are clearly not syntenic.

10.1128/mbio.02868-22.4 FIG S4 Analysis of the repeats of the LEA880-like sequence within the *Eimeriidae*. (A) Sequence logo representation of the aligned six repeats for each species (gene accession numbers in parentheses), indicating some resemblance to that of the respective *Toxoplasma* logo (top). (B) Helical wheel representation of the individual repeats of TgLEA880, indicating their amphipathic nature. Nonpolar and hydrophobic residues are colored in gray and yellow, respectively. Numbers refer to the designated area in Fig. S5. (C) The same analysis as in B, performed for the E. tenella LEA880-like protein repeats as a representative example. Download FIG S4, TIF file, 3.1 MB. Copyright © 2023 Arranz-Solís et al. 2023 Arranz-Solís et al. https://creativecommons.org/licenses/by/4.0/ This content is distributed under the terms of the Creative Commons Attribution 4.0 International license.

For the other two LEA proteins, two repetitive patterns each could be observed (Fig. S3A, C). A search for domains in the Conserved Domain Database (CDD), Pfam, and Interpro databases did not result in hits for TgLEA850, -870, or -880. For TgLEA860, on the other hand, homology for Interpro domain PTHR47372:SF11 subfamily: DAUER UP-REGULATED-RELATED was found, a domain present in other plant LEA proteins (http://www.pantherdb.org/panther/family.do?clsAccession=PTHR47372).

**FIG 2:**
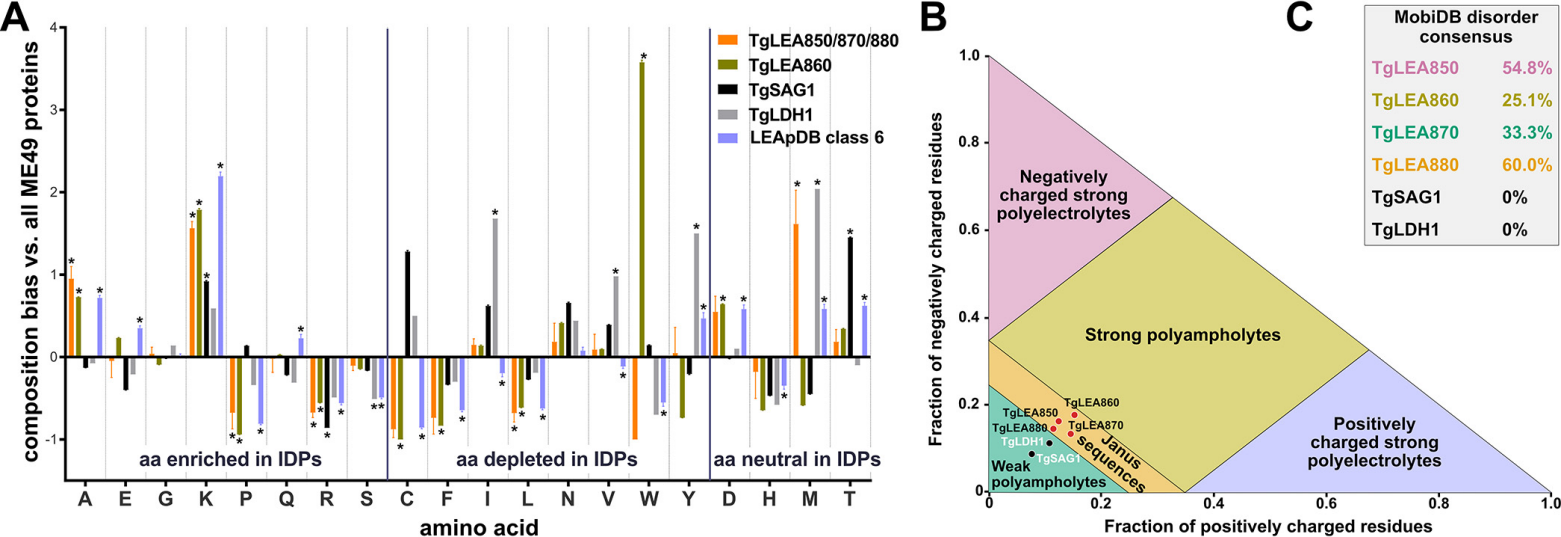
Bioinformatic analyses of TgLEA8x0 validate their assignments as IDPs. (A) Compositional bias of aa of TgLEA850, -870, and -880 as a group and TgLEA860 individually (due to its extreme W content) in comparison to the whole T. gondii proteome. TgSAG1 and TgLDH1 (non-IDP proteins), as well as a subset of proteins from LEApDB grouped into class 6, are included for comparison. *, *P* ≤ 0.002500. (B) Das-Pappu phase diagram of TgLEA8x0, including TgSAG1 and TgLDH1 as controls. (C) Disorder consensus values from MobiDB for indicated proteins.

Since, in general, LEAs are IDPs, we analyzed TgLEA8x0 for IDP features using different bioinformatic tools. IDPs have a biased aa composition, indicated by an enrichment in charged, hydrophilic residues and small aa like Ala, Ser, and Gly, whereas more hydrophobic aa are depleted in IDPs (56, 57). Compositional bias was evident for many, but not all, aa in TgLEA8x0 compared with 8,284 *Toxoplasma* proteins (Fig. 2A), including TgSAG1 and TgLDH1, two non-IDP proteins of known globular structure (58, 59). Aa composition of 297 class 6 proteins from LEApDB, which have a particular bias in alanine (50), follows the same pattern of bias, reinforcing the assignment of TgLEA8x0 to class 6.

We determined the position of TgLEA8x0 within the Das-Pappu phase diagram (60), which shows the fraction of positive versus negative charges of the proteins. As shown in Fig. 2B, they fall into the region of so-called Janus sequences, whereby their conformation could be either collapsed or expanded, depending on surrounding factors, such as salt concentration, water content, or protein interactions (60). This is consistent with TgLEA8x0 being IDPs. The globular proteins TgSAG1 and TgLDH1, by contrast, are assigned as weak ampholytes in this diagram. MobiDB, a database of protein disorder and mobility annotations, which provides a consensus percentage for the disorder calculated from several individual predictors of intrinsic disorder (61), also indicated a high percentage of disorder in TgLEA8x0, as opposed to TgSAG1and TgLDH1 (Fig. 2C). We finally confirmed these assignments using other recently described algorithms, including flDPnn (62) and Metapredict, a consensus predictor of intrinsic disorder (63) and which also calculates pLDDT, another score of disorder based on AlphaFold2’s structure prediction algorithm (64) (Fig. S5). Again, TgSAG1 and TgLDH1 showed little evidence of unstructured regions compared to TgLEA8x0, all of which displayed disorder propensities above the threshold along their entire sequences.

10.1128/mbio.02868-22.5 FIG S5 Disorder prediction of TgLEA8x0, TgSAG1 and TgLDH1 by different algorithms. Disorder score (0 to 1) for each aa is plotted for flDPnn (blue), Metapredict (red) and pLDDT (green). The black line indicates six or more consecutive residues with a positive disorder value on MobiDB by more than 50% of the predictors. Stippled lines indicate respective cutoff values for flDPnn and pLDDT in their respective colors. The latter is inversely correlated with disorder. Download FIG S5, TIF file, 1.5 MB. Copyright © 2023 Arranz-Solís et al. 2023 Arranz-Solís et al. https://creativecommons.org/licenses/by/4.0/ This content is distributed under the terms of the Creative Commons Attribution 4.0 International license.

### LEA8x0 genes from *Sarcocystidae* are subtelomeric and putative *de novo* genes.

The limited sequence homologies of the *Sarcocystidae* LEA gene products with proteins from other organisms, mostly confined to aa in their amphipathic repeat regions (data not shown), raises the question about the origin of the former. We realized that in T. gondii, N. caninum, and *B. besnoiti*, for which the available genome information includes the respective telomeric end, the *LEA* genes are subtelomeric, being located between 6.6 kb (N. caninum) up to 201 kb (T. gondii) upstream of the telomer (Fig. 1A) (65). Note that the annotated syntenies on ToxoDB (release 59) are not entirely correct (Fig. S2B). Importantly, strict synteny is observed in *Toxoplasmatinae* (T. gondii, *H. hammondi*, N. caninum, and *B. besnoiti*) up to LEA850, whereas it breaks down for *C. suis* (*LEA870* on another contig) and more so for *S. neurona* where the genes for *LEA850* and *LEA880* are direct neighbors, and no orthologs for *LEA860* and *LEA870* can be found (Fig. 1A, Fig. S2B). Species of the *Eimeriidae* family do not show any closely related sequences in such an arrangement. Such loss of synteny within relatively closely related species is one of the hallmarks of *de novo* gene birth (66). Moreover, subtelomeric regions are known to be sites where *de novo* genes have been typically reported to emerge and cluster (67), and we propose that the *LEA* genes in *Sarcocystidae* arose *de novo* rather than being acquired by horizontal gene transfer (detailed in the Discussion).

### Experimental assessment of TgLEA8x0 as IDPs.

**FIG 3:**
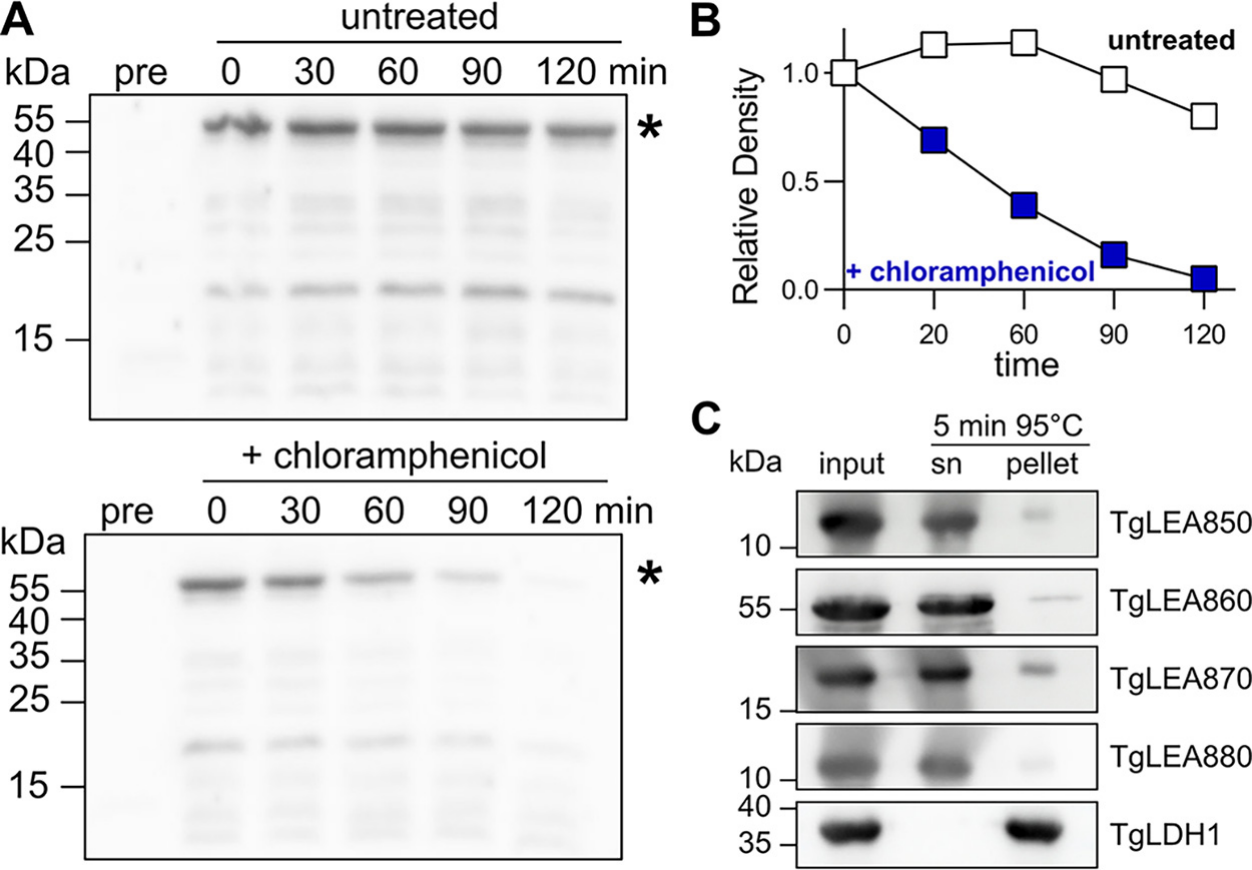
Stability assessment of TgLEA8x0. (A) Western blot with anti-6×His tag antibodies of bacterial lysates expressing TgLEA860 plus/minus chloramphenicol treatment after induction for the indicated times, or before induction (pre). *, Predicted full-length protein of MW 53 kDa. The experiment was performed twice, with similar outcomes. (B) Quantification of the protein signal (*) in panel A. (C) Western blot with anti-6×His tag antibodies of purified TgLEA8x0 and TgLDH1 before (input) and after heating for 5 min at 95°C. After the treatment, samples were separated into supernatant (sn) or pellet. Bands observed in the pellet fraction correspond to heat-denatured, and thus precipitated, proteins. The experiment was performed thrice, with similar outcomes.

To further characterize TgLEA8x0, we recombinantly expressed them in E. coli and purified them by Ni-NTA affinity chromatography. They were readily purified as fairly stable proteins, although showing some degradation over time, a fact most notable for TgLEA860. We noticed a multitude of degradation products by Western blotting almost immediately after purification, still containing the C-terminal 6×His tag, which was pronounced upon storage (not shown). This enhanced proteolysis was also shown most dramatically for TgLEA860 by an *in situ* protein turnover assay in E. coli using antibiotic chase with 40 μg/mL chloramphenicol, followed by detection of the protein in cell lysates by Western blotting (Fig. 3A and B). The fast disappearance of TgLEA860 in chloramphenicol-treated cells compared to those where protein synthesis commenced indicated a “degradation by default” behavior of TgLEA860 (68). It is well established that IDPs or proteins containing intrinsically disordered regions (IDRs) are more susceptible to proteases, a feature which can serve as an experimental characteristic for IDPs (69). This was also evident for all 4 TgLEA8x0 proteins, being highly sensitive to trypsin or thermolysin digestion compared to the globular protein lysozyme (data not shown).

**FIG 4:**
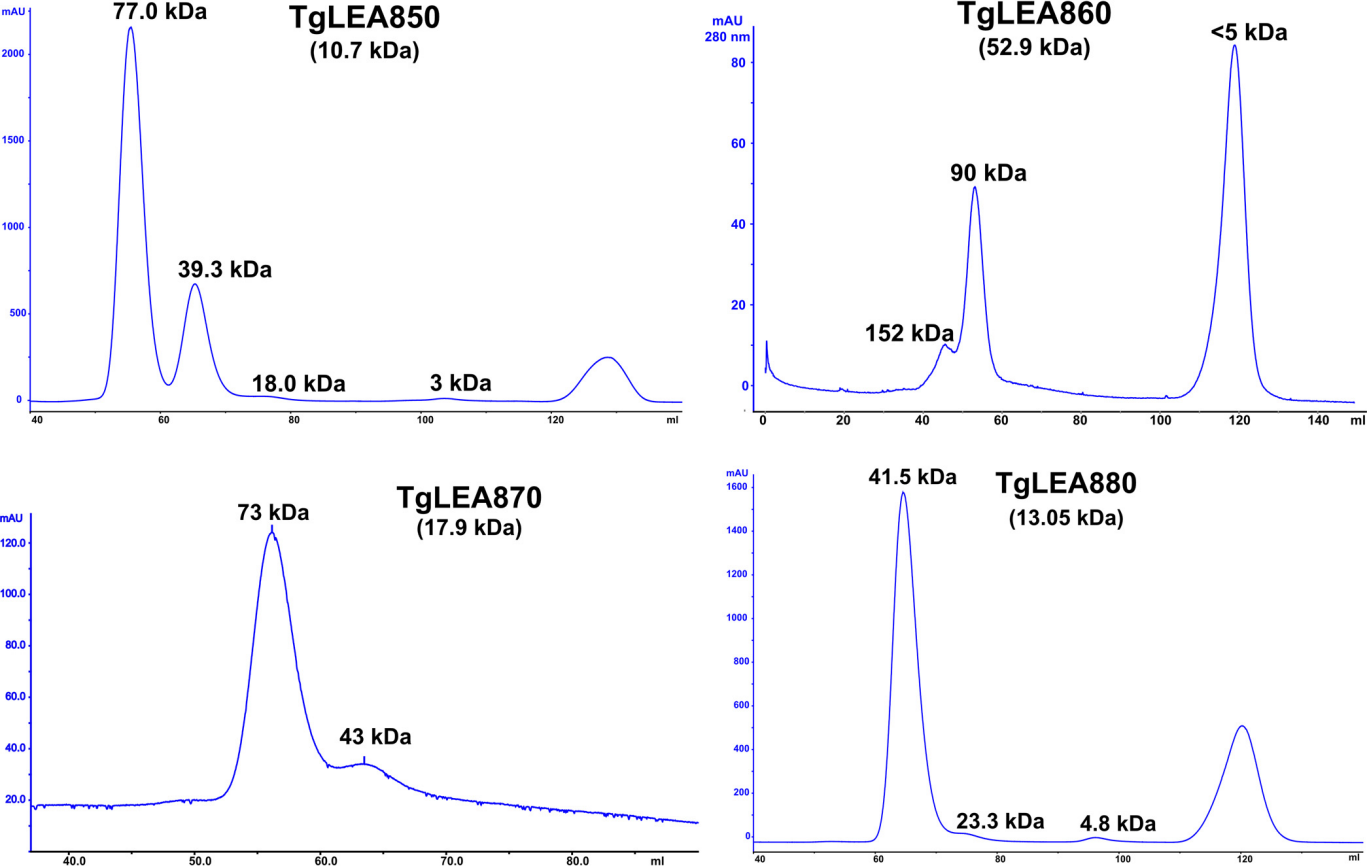
Analytical size exclusion chromatography of purified TgLEA8x0. Molecular weights (MW) at the top of the peaks are based on the calibration curves, indicating a larger hydrodynamic radius than predicted from the calculated MW of the individual protein (in parenthesis). Each experiment was performed twice, with similar outcomes.

IDPs are also known for their resistance to aggregation due to denaturation by heat treatment (69). After purification, when TgLEA8x0 were incubated for 5 min at 95°C and analyzed by SDS-PAGE and Western blot, it was apparent that, in contrast to TgLDH1 which precipitated quantitatively, all four TgLEA proteins remained in solution (Fig. 3C). In fact, this property could be used to deplete the purified proteins from minor impurities (not shown). Another hallmark of IDPs is their aberrant behavior during size exclusion chromatography (SEC) due to their less compact conformation compared to globular proteins of similar size, thus possessing a larger hydrodynamic radius (70). This should not be confused with a larger molecular weight when IDPs are separated on a SEC column that has been calibrated with globular proteins, as it is commonplace. Accordingly, TgLEA8x0 did not show peaks at the expected respective elution volume of a globular protein of that size but rather eluted much earlier (Fig. 4), consistent with larger hydrodynamic radii under physiological buffer conditions.

**FIG 5:**
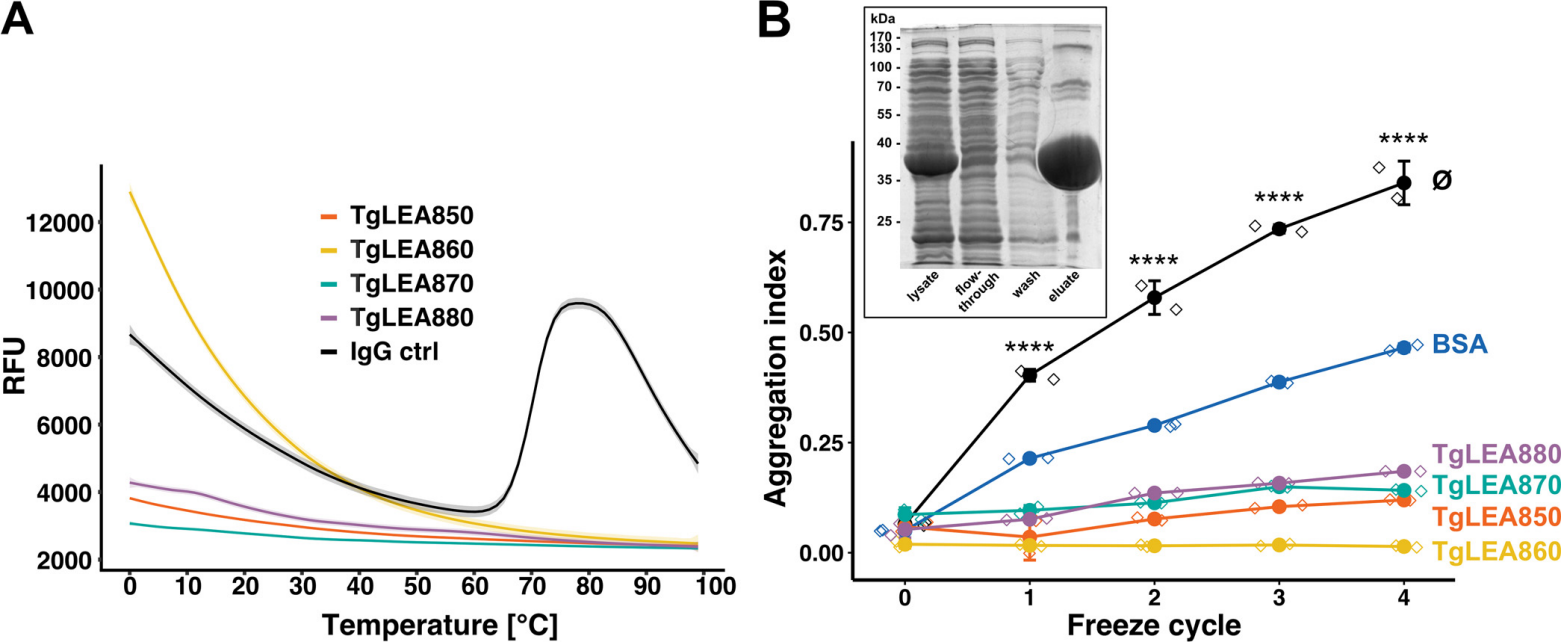
Analysis of conformational changes of TgLEA8x0 and their cryoprotective effects on TgLDH1 *in vitro.* (A) Differential scanning fluorimetry of purified TgLEA8x0. IgG served as a globular control protein. RFU, relative fluorescent units. Shaded area: 95% CI of SEM; *n* = 4 experiments, no replicates. (B) Prevention of TgLDH1 aggregation by TgLEA8x0 after repeated freeze-thaw cycles at a 1:25 molar ratio. Ø indicates TgLDH1 without any additive. BSA served as control for a cryoprotectant. *n* = 2 experiments, no replicates. Means ± SD are shown. Diamond symbols depict individual values. ****, *P* < 0.001; comparing any of the additives to TgLDH1 Ø, using two-way ANOVA with Dunnett’s multiple-comparison test. The inserted figure on the top left corner of panel B corresponds to the purification of recombinant TgLDH1 purified by affinity chromatography in a single step to the level of purity shown in lane “eluate” (>95%).

We intended to analyze protein conformational changes in TgLEA8x0 by differential scanning fluorimetry (aka thermal shift assay) (71), which is based on the binding of a fluorescent dye to hydrophobic patches within a protein structure which are exposed, and thus accessible, to the dye upon heat treatment. An ideal globular protein will show an increase in this extrinsic fluorescence close to its melting temperature, followed by a sharp decline due to denaturation further along the temperature gradient (Fig. 5A; IgG control). In contrast, nonideal folded proteins, including IDPs, show no sigmoidal transition curves upon denaturation and/or high fluorescence at room temperature. Surprisingly, TgLEA8x0 did not show signs of fluorescence changes that would be consistent with dye binding and thus significant structural changes upon heating, consistent with other small IDPs (72) (Fig. 5A). The high intrinsic fluorescence of TgLEA860 seen at lower temperatures is probably due to its very high tryptophan content (Table S1).

### TgLEA8x0 function as cryoprotectant for parasite lactate dehydrogenase.

A major function of LEAs in other organisms is protection from temperature stresses. In initial experiments we investigated the protective effect of TgLEA8x0 on porcine lactate dehydrogenase (pLDH), a frequently used reporter for this purpose. Aggregation of pLDH upon repeated freeze-thaw cycles can serve as an indicator of protein denaturation upon cold stress, and we observed a substantial reduction in the aggregation index (AI) for all four TgLEAs at a molar ratio (pLDH/TgLEA8x0) of 1:25 or higher (data not shown), with an AI of 1, indicating complete aggregation and 0 complete solubility. Since LDH transcripts and protein are highly abundant in *Toxoplasma* oocysts (47, 48) we assessed whether TgLEA8x0 have a similar effect on a potential physiological substrate of oocysts, TgLDH1. As shown in Fig. 5B, this is in fact the case, as TgLEA860 showed the most pronounced effect, preventing the aggregation of TgLDH1 by almost 100%, even after four freeze-thaw cycles. Bovine serum albumin (BSA), a known cryoprotectant, was much less efficient at the same 1:25 ratio (~45%).

### TgLEA860 and TgLEA880 expression improves recovery of E. coli after cold-induced stress.

**FIG 6:**
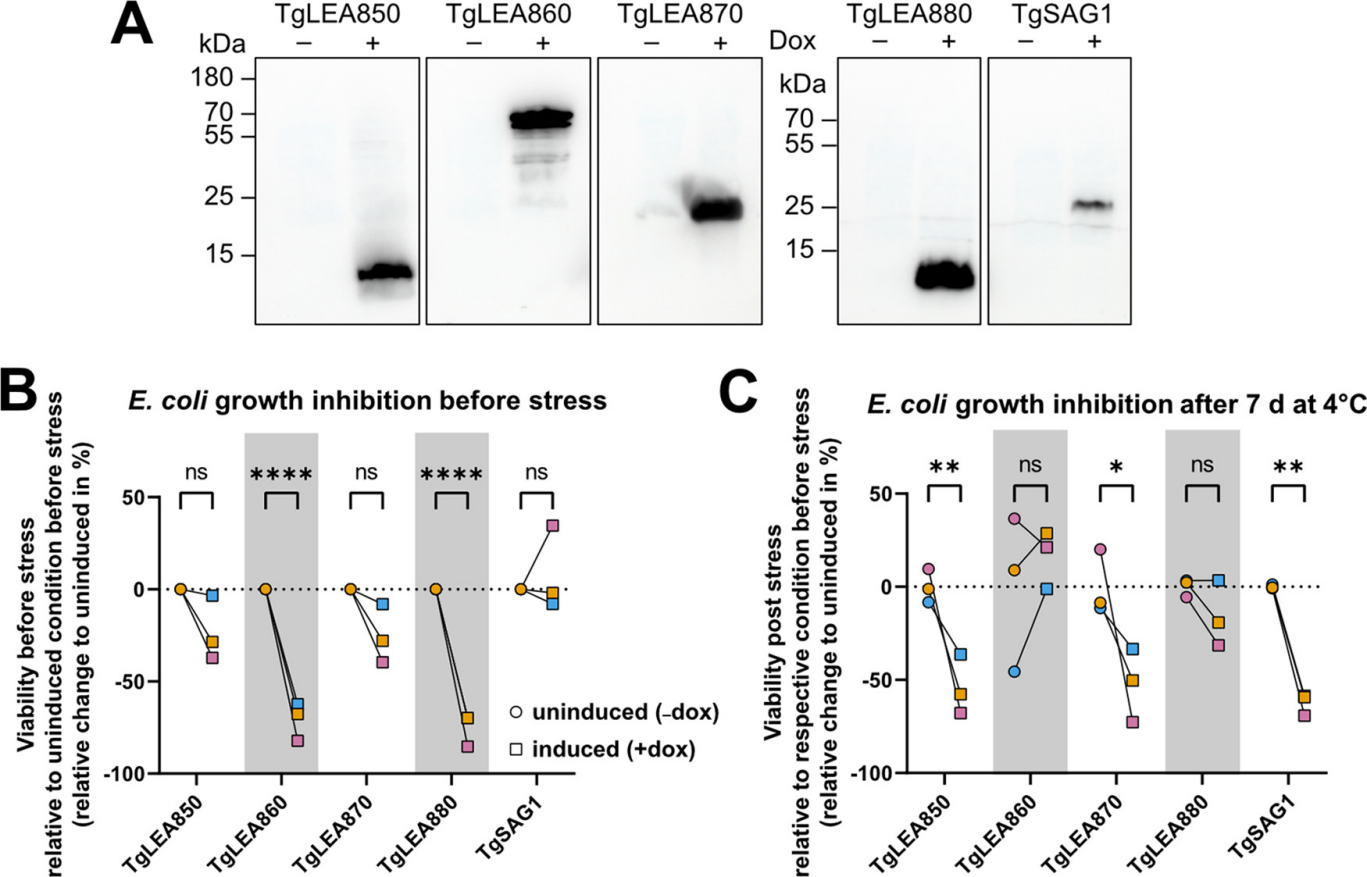
Cryoprotective effects of TgLEA8x0 *in situ* in bacteria. (A) Western Blots showing the expression of TgLEA8x0 proteins or TgSAG1 in the E. coli strain BW25113 1 h after induction. Note that expression was only observed in the presence of 200 ng/mL doxycycline. Similar amounts of cell lysates were separated by SDS-PAGE, and proteins were detected with anti-6×His tag antibody. (B and C) One hour after induction, bacteria were subjected to 4°C cold stress for 7 d. Viability of identical samples was determined by growth-based viability assay before and after cold stress (see Materials and Methods). The means of three biological replicates are shown as percentage difference compared to the uninduced samples (matching colors indicate the same experiment). Two-way ANOVA with Sídák multiple-comparison test was performed. (B) Viability of samples before cold stress was calculated based on viability calibration curves of uninduced samples before stress. ****, *P* < 0.0001. (C) Viability of samples post stress was calculated based on calibration curves of the respective experimental condition (uninduced or induced) before stress. Growth inhibition was significant for TgLEA850 (*P* = 0.0092), TgLEA870 (*P* = 0.0120), and SAG1 (*P* = 0.025), but not significant (ns) for TgLEA860 and TgLEA880 (*P* = 0.82 and *P* = 0.84, respectively), suggesting mild cryoprotective effects.

We next sought for an *in situ* model of protection from cold-induced stress, and overexpression of LEA proteins in E. coli has been repeatedly used for this purpose (53, 73). All four TgLEAs were expressed in the E. co*li* BW25113 strain (74) under a strongly regulated Tet promoter, resulting in no detection of the proteins under noninducing conditions (Fig. 6A). The globular protein TgSAG1 was not expected to show cryoprotective effects and served as a control. After protein expression for 1 h, doxycycline was removed, and bacteria viability was determined either immediately (pre-cold shock) or after 7 d of storage at 4°C (post-cold shock) using a growth-based viability assay in microtiter plates (75) (see Materials and Methods for details). Doxycycline itself was not toxic to the bacteria at the concentrations used in our experiment (200 ng/mL, data not shown), but the induced overexpression had a varying negative impact on the bacterial growth rates (Fig. 6B; Fig. S6C–E). The induction of TgLEA860 and TgLEA880 significantly inhibited E. coli growth before exposure to stress. After exposure to 4°C for 7 d, viability of bacteria with protein induction was overall lower than uninduced samples (Fig. 6C; Fig. S6C–E). Growth inhibition was significant for TgLEA850, TgLEA870, and TgSAG1, whereas this was not observed for TgLEA860 and TgLEA880, even though overexpression of these two proteins itself had a negative impact on growth under nonstress conditions (Fig. 6B; Fig. S6C–E). Taken together, this implies a beneficial role of TgLEA860 and TgLEA880 for survival at low physiological temperatures in a surrogate host compared to the other tested proteins.

10.1128/mbio.02868-22.6 FIG S6 Growth curves of E. coli growth-based viability assay. (A) Schematic drawing of experimental procedure. (B) Schematic drawing and example data analysis for representative growth curves of induced TgLEA850 sample. The top left graph shows growth curves of the dilution series measured before exposure to cold stress. Dilution “A” corresponds to 100% viability. Fractional cycle numbers (C_t_) were determined as described in Methods and a calibration curve was determined by linear regression (top right). Bottom left graph shows growth curves for identical sample (dilution “A”) post 7 d at 4°C. The viability of each sample was measured using the calibration curve and is shown in the bottom right graph. (C to E) Growth curves obtained for all 3 biological replicates. Optical density was measured at 600 nm every 550 s (1 cycle). Grey curves represent the serial dilution measured before stress and red curves the sample of dilution “A” post 7 d at 4°C. Note that the curves show regrowth of the samples after they have been induced/not induced for 1 h. No doxycycline was present during the measurement of these growth curves. Download FIG S6, PDF file, 0.5 MB. Copyright © 2023 Arranz-Solís et al. 2023 Arranz-Solís et al. https://creativecommons.org/licenses/by/4.0/ This content is distributed under the terms of the Creative Commons Attribution 4.0 International license.

### Knocking out the cluster of four *LEA* genes in a cat-compatible ME49 strain.

In order to investigate the function of *TgLEA* genes, we used a cat-compatible ME49 strain (ME49 Δ*hpt* luc^+^) to knock out the four *TgLEA* genes located in the aforementioned gene cluster (Fig. 1A). Notably, based on the transcriptomic data available on ToxoDB, there are no other transcripts described in the *TgLEA* cluster. To generate this *TgLEA* cluster knockout strain (ME49 Δ*leac*), a plasmid containing the CRISPR/Cas9 cassette and two gRNAs targeting the 5′ end of the first gene (*TgLEA850*) and the 3′ end of the last gene (*TgLEA880*) was cotransfected together with a PCR-amplified repair template containing a copy of the *HPT* gene flanked by upstream and downstream *TgLEA* homology regions. Using the primers laid out in Fig. S7A, several clones tested positive by the diagnostic PCRs, indicating the complete removal of the four genes and the substitution of the whole cluster with the transfected HPT repair template by double homologous recombination. Clone no. 3 was selected for further experiments (Fig. S7B).

10.1128/mbio.02868-22.7 FIG S7 Strategy to disrupt the LEA cluster (LEAc). (A) a diagram depicting the endogenous locus with the 4 LEA genes (TGME49_276850, 276860, 276870 and 276880) and the repair template used for the CRISPR/Cas9-induced double homologous recombination. The positions of gRNAs used for the LEAc disruption (gRNA5 and gRNA3) are indicated by black arrows. Primers F1, R1, F2, R2, F3 and R3 (readers are referred to **Table S3** for further details) binding sites are depicted by small red, brown and green arrows, and PCR amplicon lengths indicated by dashed arrows and number of bases, either in bp or kb units, are shown. (B) Agarose gels with examples of PCRs used to detect clones with disrupted LEAc. The identity of the samples is shown at the top of each lane. Primers used and length of the expected amplicon for each PCR is indicated at the bottom of each gel. Numbers on the ladder lanes show the most relevant band sizes, in kb, as a reference. # indicates clone number, KO: knock-out; mix pop: mixed population (population of transfected and selected parasites immediately before limiting dilution); WT: wild-type; NEG: negative. Clone no. 3 was selected for subsequent experiments. Download FIG S7, TIF file, 4.3 MB. Copyright © 2023 Arranz-Solís et al. 2023 Arranz-Solís et al. https://creativecommons.org/licenses/by/4.0/ This content is distributed under the terms of the Creative Commons Attribution 4.0 International license.

### ME49 Δ*leac in vivo* virulence and cyst formation remains unaltered.

**FIG 7:**
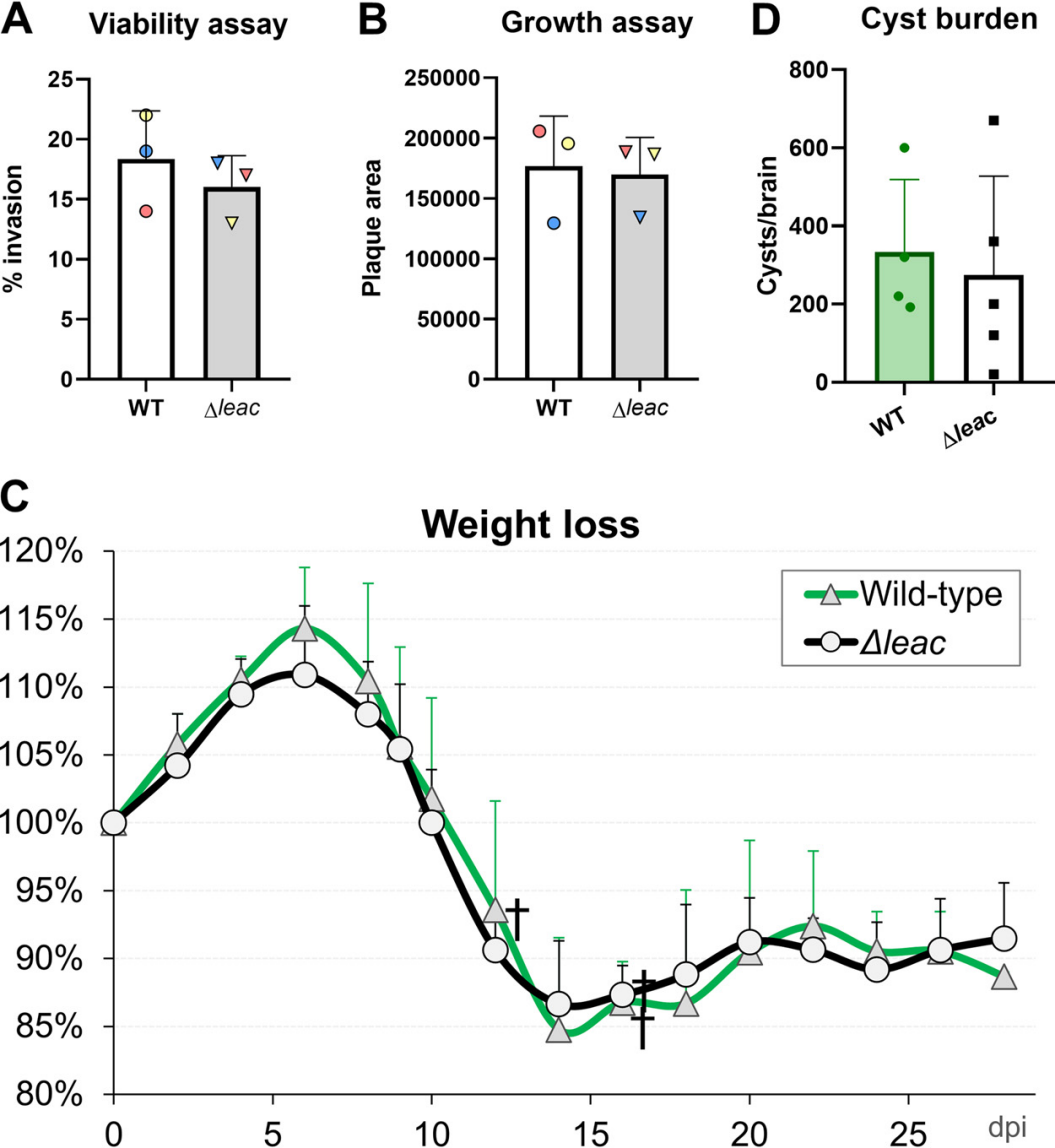
*In vitro* and *in vivo* assessment of the ME49 Δ*lea*c strain. (A and B) HFFs were plated in 24-well plates and infected with 200 parasites of the ME49 knockout (Δ*leac*) and wild-type (WT) (Δ*hpt*) strains for 7 days, after which (A) the number of plaques and (B) plaque areas were measured. Experiments were performed in triplicates, and for each strain each dot represents the mean percentage of invasion of 4 individual wells (A), or the average plaque area of at least 25 plaques from triplicate wells (B). Statistical analysis was performed by two sample Student's *t* test. Data are represented as mean ± standard deviation. (C and D) Six CD1 mice were infected with 5,000 freshly harvested tachyzoites intraperitoneally with the ME49 Δ*lea*c and ME49 Δ*hpt* (WT) strain. (C) Survival and percentage of weight decrease was recorded for each group. † indicates mouse death events. Dpi: days postinfection. (D) Brains were collected at 4 weeks postinfection from each surviving mouse, and the number of cysts per brain estimated by DBA staining. Bars indicate the average number of cysts for each group, while individual dots represent each mouse cyst count. Error bars represent the standard deviation for each group.

The *in vitro* phenotype score (which can be found on ToxoDB) for the four *TgLEA* genes predict that these are dispensable (0.26, −0.95, 0.59, and 0.61 for *TgLEA850* to *TgLEA880*, respectively). As expected, the ME49 Δ*leac* strain showed similar *in vitro* growth compared to wild-type parasites in terms of invasion rate and plaque area formation (Fig. 7A and B). Next, we set out to test the *in vivo* virulence, cyst formation, and oocyst production in the knockout strain. Thus, using parasites with a low passage number after transfection (<10), we infected mice intraperitoneally with 5,000 tachyzoites of the ME49 Δ*leac* strain in parallel with the parental strain (ME49 Δ*hpt*, here called WT) as a control. Both parasites showed a similar pattern of infection, with mice decreasing weight in the first week postinfection (p.i.) and recovering after the second week p.i. In the WT, 2 out of 6 mice died, while 1 out of 6 mice succumbed in the ME49 Δ*leac* group (Fig. 7C). These results are in line with the average virulence of this ME49 strain in CD1 mice that we have observed in several experiments (0 to 50% mortality rate at a dose of 5,000 tachyzoites IP, unpublished observation). No statistical differences in terms of weight decrease or survival with the parental strain were detected. After 4 weeks p.i., all surviving mice were sacrificed, their brains collected, and cysts counted by Dolichos biflorus agglutinin (DBA) staining. The number of cysts per brain was variable between individuals but similar within groups, with an average cyst yield of 333 and 274 cysts per brain for the WT and Δ*leac* strains, respectively (Fig. 7D). Overall, our results suggest that there are no apparent defects in the virulence and cyst formation in the ME49 Δ*lea*c strain.

### LEA knockout oocysts do not show apparent morphological or sporulation defects.

**FIG 8:**
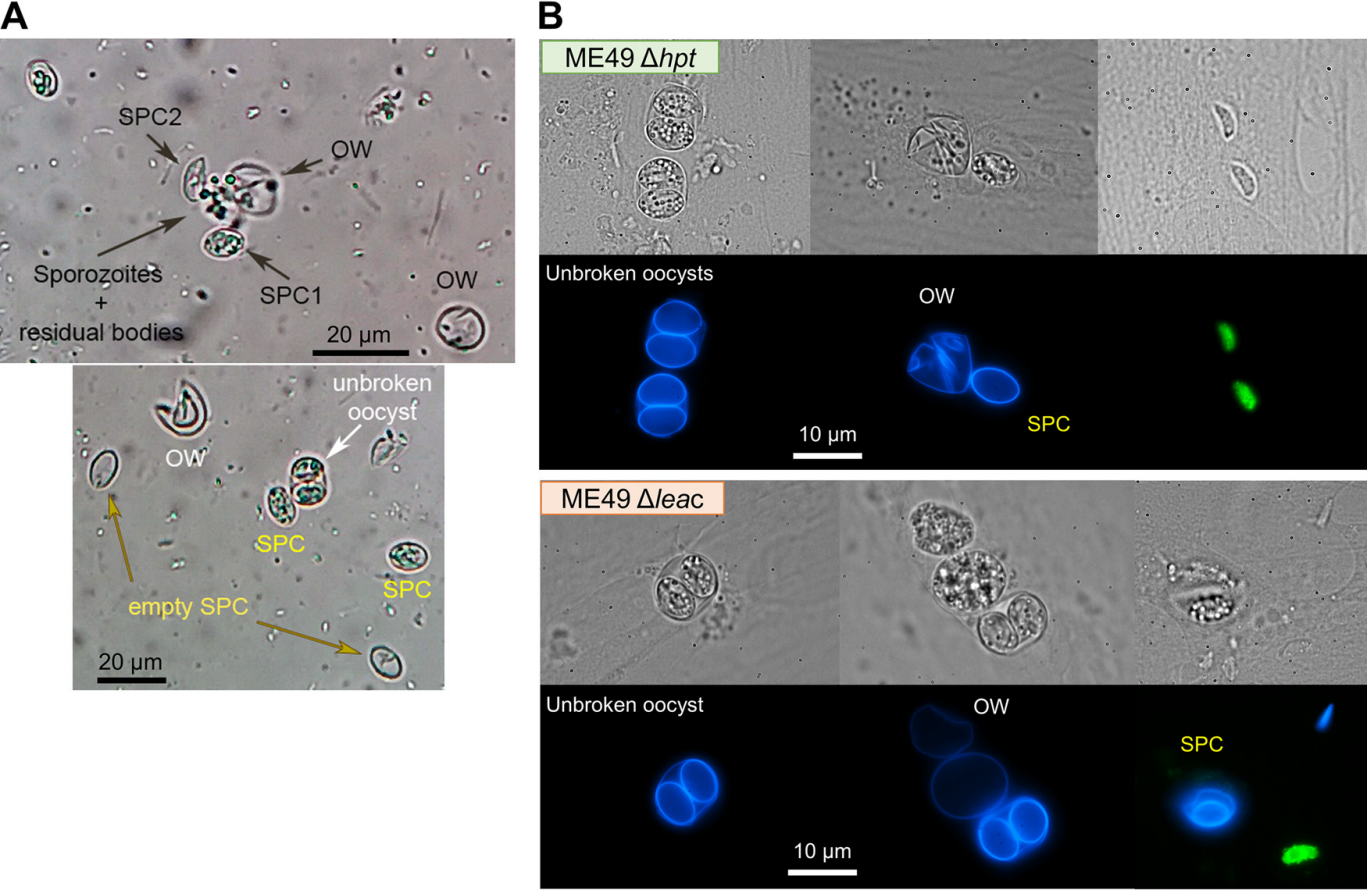
Excystation and morphology of ME49 Δ*lea*c and ME49 Δ*hpt* oocysts. Sporulated oocysts were excystated as described in the Material and Methods section. (A) Representative pictures taken at 40× after the glass bead treatment, showing different stages that can be found during the process. (B) Representative immunofluorescence assay (IFA) images of intact and excystated oocysts from the ME49 Δ*hpt* (top) and Δ*lea*c (bottom) strains seeded onto confluent HFF cells for 18 to 24 h. Intracellular parasites (most likely tachyzoites) are stained in green, while the blue channel is showing UV autofluorescence from oocyst and sporocyst walls. SPC, sporocyst; OW, oocyst wall.

To assess the phenotype of the Δ*lea*c oocysts, two 6-month-old kittens were orally infected by feeding them the equivalent of 3 mouse brains containing cysts of the parental (WT) and Δ*lea*c strains, which would account for approximately 1,000 and 820 cysts, respectively (see above and Fig. 7D). Oocysts were detected in the feces of both kittens starting at day 5 p.i., recovering a similar number of purified oocysts from both animals (30 and 34 million oocysts from the WT and Δ*lea*c, respectively) after three consecutive days, and with no apparent morphological differences before sporulation. After 7 days of sporulation in 2% sulfuric acid and gentle rotation at RT, the percentage of sporulated oocysts was calculated, with a lower sporulation rate detected in Δ*lea*c oocysts compared to the WT oocysts (~65% versus ~85%). Because sporulation is a one-time event, it cannot be determined if this difference was statistically significant. Nevertheless, because previous experiments with the ME49 and M4 strains have yielded sporulation rates ranging from 60% to 90% (unpublished results), this sporulation rate is within the normal variation, a fact also observed in previous reports (31). Morphologically, WT and Δ*lea*c oocysts were indistinguishable under the microscope, and the level of excystation and invasion by plaque assay showed no differences between both strains (not shown), indicating that Δ*lea*c oocysts appear normal with no defect in excystation and/or viability of the released sporozoites (Fig. 8). Notwithstanding, more data would be needed to corroborate this information.

### *Toxoplasma* oocysts lacking *LEA* genes are more sensitive to high salinity, drought and freezing conditions.

Once we confirmed that Δ*lea*c oocysts had no morphological or viability deficiencies under normal conditions, we set out to assess the putative role of the *TgLEA* genes in environmental resistance to harsh conditions. Hence, WT or Δ*leac* sporulated oocysts were initially subjected to treatments with 50% Clorox (household bleach), high salt (~26%), 37°C, –20°C and desiccation for 24 h. This single initial trial showed that although oocysts are considered resistant to bleach, a 24-h treatment with 50% household bleach (approximately 3% sodium hypochlorite or NaOCl) is sufficient to dramatically reduce their viability, as only after adding 20,000 excystated treated oocysts, a few plaques were detected (0% and 1.6% viability in the WT and Δ*leac* strains, respectively). On the other hand, only a slight decrease in viability was observed for the temperature treatments compared to RT. Specifically, after 24 h at 37°C, 81.9% and 81% viability was recorded for the WT and Δ*leac* strains, respectively. Similarly, at –20°C, the viability percentages were 89.9% and 75% for the WT and Δ*leac*, respectively. As for the high salinity condition, the effect was more pronounced, with 72% (WT) and 50.9% (Δ*leac*) viability. Finally, when oocysts were dried out as a pellet in an open tube and left at RT for 24 h, there was a sharp decrease in viability with 6.7% and 4% in the WT and Δ*leac* strains, respectively.

**FIG 9:**
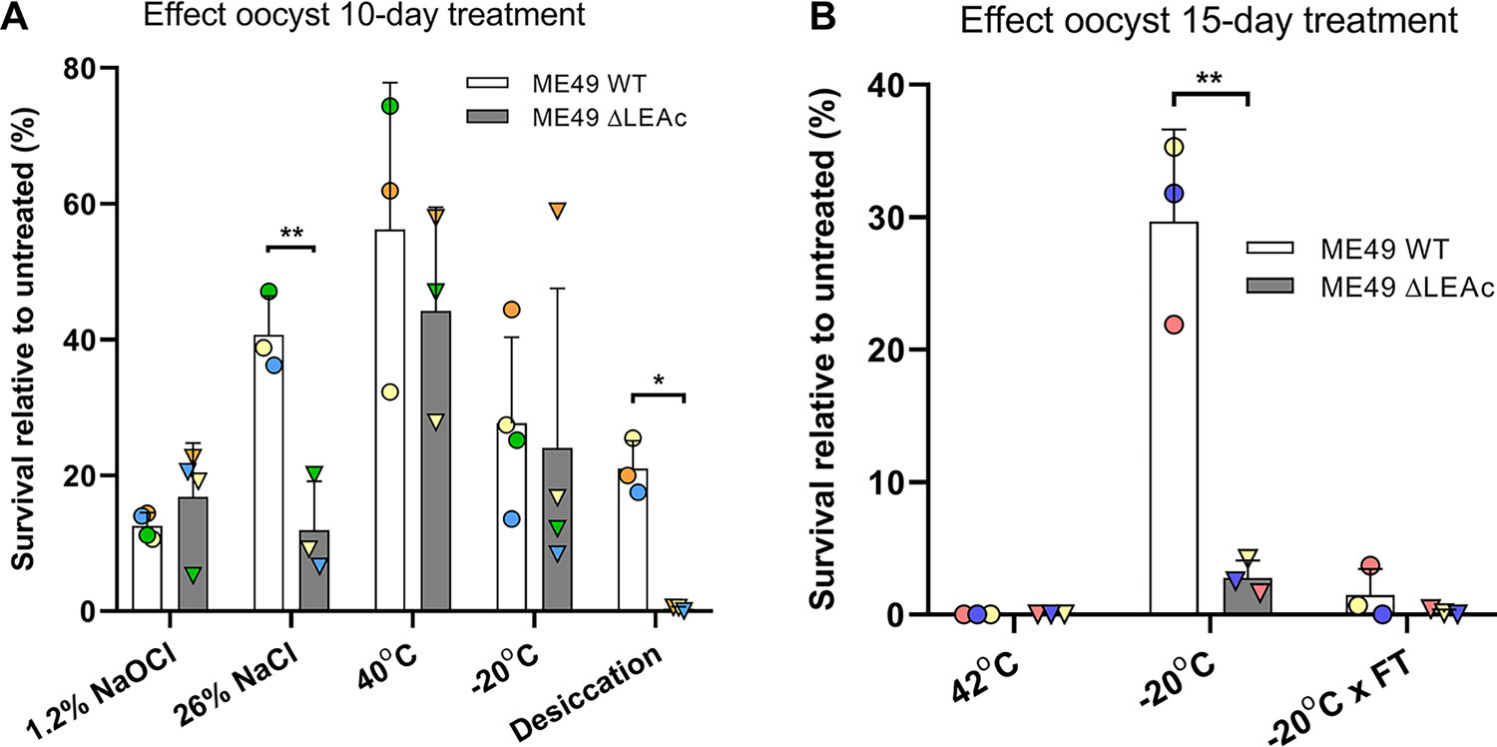
Effect of environmental treatments on oocysts. Bar graphs showing the relative survival percentage of sporozoites after diverse treatments of sporulated oocysts from the knockout (Δ*lea*c) and wild-type (WT) (Δ*hpt*) strains for 10 (A) and 15 (B) days compared to room temperature (RT). For each condition, 3 experimental replicates were performed, having 3 biological replicates in each. The percentage of excystation was calculated by dividing the number of plaques observed by the theoretical number of excystated oocysts added in the well. An average of this excystation value was calculated for each condition by combining the results obtained for each biological replicate. Subsequently, results for each condition were normalized with the respective untreated (RT) condition and are shown as percentage of theoretical maximum survival. Statistical analysis was performed by applying an unpaired *t* test followed by Welch's correction for each condition comparing the knockout (Δ*lea*c) and WT (Δ*hpt*) strains. Error bars indicate standard deviation, and statistical significance are indicated by asterisks: *, *P* < 0.05; **, *P* < 0.01.

Since this 24 h treatment was too harsh for the bleach and drought conditions, and the temperature and salt treatments had little effect, we modified the conditions by using a lower concentration of bleach (20% Clorox or 1.2% NaOCl) and leaving the dried pellet with the lid of the tube closed. In addition, to test whether *LEA* gene products have any influence on the resistance to high or low temperatures after longer periods, we increased the incubation time to 10 days, as well as the temperature to 40°C. Under these conditions, a statistically significant decrease in viability of the Δ*leac* strain compared to the WT strain was detected for the high salinity condition (12% ± 7% versus 41% ± 6%, *P* < 0.01). Similarly, the Δ*leac* oocysts were also significantly more susceptible to the 10-day desiccation treatment, with 0.3 ± 0.3% viability compared to 21% ± 4% in the WT strain (*P* < 0.05). On the other hand, the 20% Clorox, 40°C, or –20°C treatments had a similar effect on both the Δ*leac* and WT strains (Fig. 9A). Nevertheless, for the temperature treatments the Δ*leac* oocyst showed a trend toward lower viability percentages compared to WT (44% ± 15% versus 56% ± 22% at 40°C and 24% ± 23% versus 28% ± 13% at –20°C), although these differences were statistically not significant (*P* > 0.05).

Subsequently, we decided to test the temperature conditions for 15 days but increasing from 40°C to 42°C and including a treatment at –20°C where oocysts were thawed every other day, i.e., a total of 8 freeze-thaw (FT) cycles. The latter condition was added to mimic a more realistic scenario where oocysts in nature may undergo freezing conditions at night but get warmer during the day. Our results show that oocysts were not able to endure a temperature of 42°C for 15 days (at least when using a combined total number of 8,000 excystated oocysts in 3 wells), while they still withstood freezing conditions for such long times, although FT cycles had a considerable negative impact on viability (Fig. 9B). Moreover, we detected a significantly higher impact on survival in the Δ*leac* oocysts compared to the WT at –20°C (3% ± 1% versus 30% ± 7%, *P* < 0.05). This difference was also observed after the FT cycles (0.1 ± 0.2% versus 1.5 ± 2.0%), although given the low survival percentages in both groups, a statistical significance was not obtained. These results were very similar after 20 days of treatment: 2% versus 22% at –20°C and 0.1% versus 0.8% for the FT condition in the Δ*leac* and WT oocysts, respectively (*n* = 1, not shown).

In summary, *Toxoplasma* Δ*leac* oocysts were more susceptible to high salinity, desiccation and low temperature insults compared to WT. This difference was more marked when oocysts were subjected to these treatments for longer periods of time (10 to 20 days).

## DISCUSSION

In *Toxoplasma*, LEA-like proteins have been reported to be highly upregulated in developed (sporulated) oocysts compared to unsporulated oocysts (47). Their expression is also strongly induced in nonsexual stages of a strain deficient in the TgMORC protein, which is a master regulator of genes expressed in the sexual stages and oocysts/sporozoites (76). This suggests that these proteins may be an important component of oocysts and/or sporozoites. In the present work, we sought to investigate the potential role of LEA proteins in the resistance of *Toxoplasma* oocysts. To our knowledge, this is the first detailed description in any protozoan species of genes related to LEA proteins and their impact on stress tolerance *in vivo*. While all four TgLEAs possess numerous features characteristic of LEAs and IDPs, their overall low sequence similarity to other LEAs without considering other structural features makes it difficult to draw firm functional and evolutionary conclusions, as pointed out above. The observable sequence similarities, e.g., between LEA880 of the *Sarcocystidae* family and the LEA880-like gene products of the *Eimeridiiae* family, or between LEA860 and CeLEA1, can be explained by the fact that LEA-like proteins, being IDPs and often also containing amphipathic helices, dictates the use of a restricted set of certain amino acids and in a specific order, resulting in such homologies. The designation “LEA” has been used across the domains of life for many such proteins with similar primary sequence properties, which could imply some evolutionary descent by horizontal gene transfer, for example by the algal endosymbiont that gave rise to the apicoplast. While we cannot rule this out, we regard it as less likely for the LEA proteins described here, not the least due to the large phylogenetic distance and lack of shared niches between plants, nematodes, and *Sarcocystidae*. Moreover, while plants possess dozens of *LEA* genes, a recent analysis reported only one such gene for green algae, suggesting no important role for their survival in an aquatic environment (37). Instead, we propose that the *LEA* genes arose *de novo*, thus being a case of convergent gene evolution.

Emergence of new genes is generally either the result of divergence of preexisting genes via gene duplication and neofunctionalization or by *de novo* emergence from nongenic sequences by the appearance of new reading frames (77, 78). We see very little identity among the four LEA proteins and compared to other genes on the genome, and together with their syntenic presence in closely related *Toxoplasmatinae* and its stepwise loss within the *Sarcocystidae* family (Fig. 1A), it argues against the former possibility (77, 78). Loss of synteny on the genome level has been described before for the coccidia, and this analysis also described *Toxoplasma*-specific gene families located at the chromosomal ends and noted that some are expressed during sexual development and in oocysts (79). This is in line with subtelomeric regions being sites where *de novo* genes have been reported to typically occur (67) and which has been suggested to have potential significance for niche adaptation (67, 80). Moreover, random protein sequences arising by chance and being structurally disordered, have been reported to be soluble and be good progenitors for more complex proteins during evolutionary processes (81). Consistent with the function of LEAs, *de novo* genes have been found to be particularly involved in stress response, environmental adaptation, and developmental processes (66, 82, 83). The absence of introns and the repetitive nature of the *LEA* genes would be in line with the expansion of gene families at subtelomeric regions in yeast (84).

The most remarkable similarity regarding overall primary structure exists for TgLEA860 with CeLEA-1, which has been well characterized in C. elegans as a protector against desiccation, temperature, osmotic stress, and damage by UV light (51–53, 85, 86). A defining feature of TgLE860 and its homologs are the numerous tryptophan doublets (WW) (Fig. S3B). Out of 20 Trp in CeLEA-1, 16 are in the homology region with TgLEA860 with its 26 residues. Trp clusters in gamma-crystallin are known to prevent aggregation in the eye’s lens, an organ constantly exposed to damaging UV radiation (87). Given the presence of a signal peptide and thus the possibility of TgLEA860 being part of the sporocyst wall, the WW doublets might be, at least partly, responsible for an UV protective effect. On the other hand, such radiation and the resulting formation of reactive oxygen species can also occur in soil (88, 89). It could thus affect oocysts shed into the environment, and lead to intra- and interprotein cross links (90–92), thereby possibly contributing to strengthen TgLEA860’s physical properties that might be required to protect oocysts from such stresses.

In this context, most attention has been given to tyrosine (Tyr)-rich proteins as potential contributors to physical strength of the oocyst or sporocyst wall, particularly in *Eimeria* species, via the generation of dityrosine-cross-linked rigid structures (25, 93, 94). However, neither direct evidence for homologous proteins in *Toxoplasma* nor the presence of Tyr cross-links in other oocyst wall proteins of this parasite has been observed (18). Moreover, while the UV autofluorescence of oocysts is widely assumed to be due to dityrosines, genetic deletion of the two aromatic amino acid hydroxylases TgAAH1 and TgAAH2, previously suggested to be involved in dityrosine bond formation, did not result in an observable loss of autofluorescence (95). It is well known that fluorescent properties of Trp and its derivatives in proteins can overlap those of dityrosines (90–92), making it difficult to assign a specific role for autofluorescence to one aromatic amino acid (derivative) alone.

A recent report characterized the importance of the repeats in CeLEA-1 for stress resistance in this nematode (53). Strikingly, it was shown that expression of miniproteins consisting of only a few repeats was sufficient to complement the deletion of the entire CeLEA-1 protein, conferring resistance to stressors like desiccation and hyperosmolarity. While TgLEA860 and -880 also contain repetitive elements, they are clearly distinct compared to the consensus sequence from plants and nematodes, respectively, although their repetitive units can be modeled into amphipathic helices (AH), as is also the case for plant and nematode sequences. AH can interact with membranes via their hydrophobic site, which can then induce their folding. Some plant LEAs were shown to be able to protect liposomes from freeze-thaw cycle-induced damage (54). Although functionally TgLEA880 might be regarded to belong to dehydrins (LEApDB class 2), these are characterized by small distinct segments (96) rather than by repeats, as observed in TgLEA880. Phosphorylation of IDPs or IDRs is known to influence their structure and thus can change the spectrum of interacting partners (97). Whether TgLEA8x0 are subject to such posttranslational modifications is unclear but could potentially influence their functional properties. While an analysis with two predictors of phosphorylation sites (with “Disorder Enhanced Phosphorylation Predictor” developed with IDPs in mind) shows various residues on each of the four proteins to be potentially modified by negatively charged phosphate groups (Table S2), a recent proteomic study of oocysts showed no indications for such modifications in TgLEA8x0 (98). Further analysis is required to explore this issue.

LDH transcripts and proteins have been shown to be abundant in *Toxoplasma* and Cryptosporidium parvum oocysts (47, 48, 99, 100). Although TgLDH1 is mostly described as being cytosolic in tachyzoites (101) and merozoites (102), at least in extracellular tachyzoites TgLDH1 was reported to be concentrated at the inner membrane complex (103). In C. parvum, LDH1 was shown to translocate from the cytosol to the parasitophorous vacuole membrane during its intracellular development, a fact that was taken as evidence for the involvement of the enzyme in energy generation (104). Interestingly, lactate is also considered to be a potential antioxidant agent by scavenging free radicals such as O^2–^ and OH (105, 106) and/or via maintaining redox homeostasis in bacteria exposed to NO radicals (107, 108). It is therefore tempting to speculate that TgLDH1 could have a function as a scavenger despite its metabolic role. If that were to be the case, its protection from inactivation by TgLEA8x0 would be also beneficial against environmental stresses.

Our bacterial overexpression system provided some insights and could be further expanded by using different bacterial strains (e.g., desiccation-susceptible strains with mutations in genes of the glycogen metabolism [109]) and different stresses, to pinpoint potential pathways and targets of protection of individual TgLEAs. However, more parasite-specific interaction partners might be missed by this approach. Also, it will be important to determine the localization of the LEA proteins in sporozoites and identify their interacting proteins and/or subcellular sites of action. Considering the evidence we gathered regarding the cryoprotective role of TgLEA proteins *in vitro*, as well as in a surrogate *in vivo* E. coli system, we set out to investigate the effect on oocysts from a strain lacking all four LEA-like genes by assessing the sensitivity to environmental stresses compared to those derived from wild-type parasites. Our results show that TgLEA proteins are important for the resistance of oocysts to, at least, high salinity, freezing, and drought conditions. When we implemented our modified excystation protocol, the percentage of excystation (plaques/initial excystated oocysts) was much lower than that reported by Villegas et al. (110) (10 to 20% versus ~90%). Although this was expected, since we omitted the bleach pretreatment of oocysts, it is also possible that different variables, such as the parasite strain, the age of the oocysts, the reagents or the centrifugation conditions might exert an influence that may account for the differences found in the excystation efficiency. Regardless, this difference did not interfere with our ability to assess the resistance of oocysts, as it was compared to the WT under the same conditions. We also corroborated the remarkable resistance of *Toxoplasma* oocysts to a variable set of harsh environmental conditions, confirming previous observations in the literature (Table 1), even when they were exposed for a long time. Indeed, after 3 weeks at –20°C we were still able to detect surviving parasites after excystation, in accordance with previous works that reported inactivation of oocysts only after 3 to 4 weeks at –20°C (32, 111, 112). As expected, when we tested the effect of freeze/thaw cycles, which represents a condition that is more likely to be found in nature when switching from warmer days to colder nights and *vice versa*, we observed a marked decrease in oocyst survival at day 15 and 20.

A common conception is that *Toxoplasma* oocysts are unaffected by bleach, which is broadly used in laboratory environments to inactivate tissue culture-derived biohazard materials (18, 113). While it is true that bleach does not inactivate oocysts in the short term as it does with other microorganisms, we observed that, when exposed to a relatively low percentage of bleach (50% Clorox, which would correspond to ~3% NaOCl) for relatively short periods of time (24 h), oocysts are severely affected. Similar observations have been reported in previous studies (31, 110, 114) (Table 1). Although we did not further investigate the bleach effect, it is likely to be more related to the concentration rather than the incubation time, as we were able to detect viable oocysts after 10 days when decreasing the concentration to 20% Clorox (~1.2% NaOCl). Although further research is warranted, it is tempting to hypothesize that high concentrations of household bleach are likely to render *Toxoplasma* oocysts noninfective after a short time. However, a high concentration of bleach would not be a natural setup for most routine decontamination procedures. Regardless, our results indicate that TgLEA proteins are probably not involved in the resistance of oocysts to bleach. This could be expected, since bleach only affects the outer layer of the oocyst wall without disrupting the structure or permeability of the inner layer and the sporocyst wall (18, 19), and 3 out 4 TgLEA proteins are predicted not to be secreted (i.e., they likely remain inside sporozoites).

Another environmental stress we tested was desiccation. Previous studies have shown that oocysts do resist low humidity conditions to a certain extent (29, 31, 32, 112), and we confirmed this in our assays. Compared to oocysts generated from wild-type parasites, Δ*lea*c oocysts were more sensitive to drought, even after short incubation periods. This finding is in line with the role of TgLEA proteins described in plants and other organisms protecting them from desiccation and other abiotic stresses (34, 36, 53, 115). Similarly, we have confirmed the resistance of *Toxoplasma* oocysts to high salinity, which has been previously described with different concentrations of salt to mimic seawater conditions (33, 116). In our work, we observed that even a saturated salt solution (26% NaCl) barely affects oocyst viability after 10 days. However, oocysts from the Δ*lea*c strain did not show this resistance and their viability was considerably reduced compared to wild-type oocysts, indicating that TgLEA proteins are involved in resistance to high salinity.

When we assessed the effect of temperatures on oocysts, we did not observe any remarkable decrease in viability at 37°C or 40°C for 10 days. When we subsequently increased the temperature to 42°C, on the other hand, no viable parasites, either in the Δ*leac* or WT oocysts, were detected after 15 days. Since no other combination of temperature and incubation time was tested in our work, further studies are needed to assess whether *TgLEA* genes are important for the resistance to warm temperatures, like what was observed for freezing conditions. For instance, long incubation times (>3 weeks) with mild temperatures (<40°C), or shorter incubation times (<1 week) with higher temperatures (>42°C) could serve this purpose. In this sense, previous studies have shown that *Toxoplasma* oocysts can survive up to 2 days at 45°C or up to 4 weeks at 40°C (28, 31) (Table 1). Moreover, other treatments, both physical and chemical, should be tested in future assays to determine the potential role of TgLEA proteins, such as disinfectants, alcohol, formalin, UV light, or ozone, among others. Notwithstanding, it is possible that, like what we observed with bleach, TgLEA proteins do not play an important role; rather the oocyst wall structure and composition may account for the resistance to common disinfectants. As a matter of fact, the oocyst wall is rich in proteins and has an acid-fast lipid coating, which serves as a barrier to chemical stressors (24). On the other hand, because none of TgLEA proteins (except TgLEA860) have a signal peptide and are not predicted to be secreted, it could be possible that the effect of TgLEAs is exerted directly on sporozoites. In other systems LEA proteins have been shown to play roles in antiaggregation, protein stabilization, and chaperone-like activities (37). It is therefore likely that they provide protection against temperature changes, salt, and drought stress directly in sporozoites, as these LEAs are predicted to be in their cytoplasm. Since our initial assays were mainly testing the resistance of the oocysts and/or sporocysts (treatments were performed before excystation), we also investigated the effect of stressing conditions on sporozoites immediately after excystation (not shown). However, our results could not elucidate whether the effect of TgLEA proteins on oocyst resistance was related to the oocyst wall, sporocyst, or sporozoites, as there was a high variability between experiments. Future assays should be aimed at addressing and standardizing these treatments on sporozoites to obtain more consistent results. For example, excystating a higher number of oocysts and purifying only released sporozoites by filtration may render results less variable and allow to draw firm conclusions. Furthermore, testing different treatments directly on released sporocysts from broken oocysts (for example, by mechanical means such as glass beads), would confirm whether the oocyst wall is dispensable for the resistance to temperature, salinity, and humidity changes or, on the contrary, LEA proteins only constitutes an extra layer of protection against these stresses.

In summary, we show here that TgLEA proteins exert an important role in conferring resistance in Toxoplasma gondii oocysts against a variety of environmental conditions (at least high salinity, freezing and desiccation). Nevertheless, this phenotype should be corroborated in the future by repeating the performed experiments in oocysts from a complemented strain. Observing a reversion to the WT phenotype in such a strain would confirm that the deletion of the 4 *TgLEA* genes was indeed responsible for the increased susceptibility of oocysts and not because of random mutations in the genome that may occur after electroporation. Furthermore, future studies using knockout strains for each of the *TgLEA* genes alone or in combination are warranted to elucidate which one of the *TgLEA*s are needed for the resistance effect or if, on the other hand, only one of them is needed for the resistance to all or a particular stress condition.

## MATERIALS AND METHODS

### Bioinformatic analyses of protein structures and features.

BLAST and DELTA-BLAST searches at NCBI were used to find similar proteins. Other databases queried with the primary sequences included Pfam (now at https://www.ebi.ac.uk/interpro), OrthoMCL DB (https://orthomcl.org) and LEApDB (50). Smith-Waterman local alignment of sequences was performed with EMBOSS WATER (https://www.bioinformatics.nl/emboss-explorer) and multiple sequence alignment using MAFFT (117). Alignments were manually adjusted where necessary and visualized with Jalview (118). Repeats were detected using the TRUST repeat detection server (https://www.ibi.vu.nl/programs/trustwww/) using default options (119); RADAR at EBI (https://www.ebi.ac.uk/Tools/pfa/radar/) or ETANDEM for DNA (https://www.bioinformatics.nl/emboss-explorer/). The resulting sequences were manually aligned. Amphipathic wheel visualizations of the repeats were performed using the HELIQUEST web server (http://heliquest.ipmc.cnrs.fr/cgi-bin/ComputParams.py) (120). For all four *TgLEA*s, the sequences available for the 64 T. gondii strains were downloaded from ToxoDB (https://toxodb.org) (121), aligned, and then represented as sequence logos with WebLogo (http://weblogo.threeplusone.com) (122). Phosphorylation site predictions were performed with Disorder Enhanced Phosphorylation Predictor (DEPP) (http://www.pondr.com/cgi-bin/depp.cgi) and NetPhos-3.1 (https://services.healthtech.dtu.dk/service.php?NetPhos-3.1). Only consensus sites that were above the respective cutoffs of each algorithm are shown in Table S2.

Compositional bias of amino acids (aa) in the LEA proteins was analyzed using CompositionProfiler (http://www.cprofiler.org/cgi-bin/profiler.cgi) (57) with 10,000 bootstrap iterations against 8,284 annotated TgME49 proteins (9) as background sample. Raw data were then visualized using Prism (GraphPad) version 9.0.

Aa frequencies and grand average of hydropathicity (GRAVY) values for each of the 8,284 annotated T. gondii proteins were analyzed using ProtParam from the BioPython package (123). In addition to the charge-hydropathy scale of Kyte-Doolittle (GRAVY-KD), the scale of Huang et al. (124) was also implemented (GRAVY-IDP), which provides a better scale for identifying protein disorder (Table S1).

Intrinsic disorder prediction was done using four metrics. First, for each sequence, we extracted from MobiDB (https://mobidb.bio.unipd.it/) (61), the binary value for each aa being annotated as disordered (1) by at least 50% of the used algorithms (example query: https://mobidb.bio.unipd.it/api/download?acc=A0A125YWT2&projection=prediction-disorder-th_50,sequence,acc&format=fasta). For graphical representation of longer disordered consensus regions along the aa sequence, we then determined regions with consecutive six or more residues having a value of 1. The other predictors were used online to obtain the raw data: the deep neural network-based algorithm flDPnn (http://biomine.cs.vcu.edu/servers/flDPnn/) (125) and the consensus predictor Metapredict (https://metapredict.net/) (63). The latter also provides the calculated pLDDT score, a per-residue estimate on the expected reliability of AlphaFold2’s confidence in its model. It is assumed that a score of <0.5 is a fairly strong predictor of disorder (64). All the individual data were then combined in a single graph per protein using R package ggpubr 0.4. The Das-Pappu phase diagram, which provides an approximation of conformations an IDP might have, was calculated online with CIDER (http://pappulab.wustl.edu/CIDER/) (126).

### PCR and sequencing.

Genomic (g)DNA was extracted from parasites for screening purposes by using DNAzol reagent (Invitrogen, ref. 10503027). All PCRs were conducted by using either Q5 Hot Start High-Fidelity DNA polymerase (New England Biolabs, cat. no. M0494S) for cloning purposes, or MangoMix (Meridian Bioscience ref. BIO-25034) for diagnostic/screening purposes. All PCR conditions were designed following recommendations by NEB TM calculator tool. When necessary for downstream applications (Gibson assembly, cloning, sequencing, etc.), PCR products were purified either directly with the DNA clean and concentration kit (Zymo Research, 11-304C) or after gel excision with the Qiaquick gel extraction kit (Qiagen, 28704). Gibson assembly constructions, plasmids, and PCR products from genes/regions of interest in mutant parasites were assessed by Sanger sequencing using the primers listed in Table S3, and sequences were analyzed by using BioEdit sequence alignment editor version 7.0.5.3 (127).

10.1128/mbio.02868-22.10 TABLE S3 Primers used in the present study. Download Table S3, DOCX file, 0.02 MB. Copyright © 2023 Arranz-Solís et al. 2023 Arranz-Solís et al. https://creativecommons.org/licenses/by/4.0/ This content is distributed under the terms of the Creative Commons Attribution 4.0 International license.

### Expression constructs.

For large-scale expression and purification, the coding sequences of TgLEA850, -870, and -880 were codon-optimized and custom synthesized as fusions with a C-terminal 6×His tag in the IPTG-inducible expression vector pQE90S (Qiagen) by GenExpress (Berlin, Germany). *TgLEA860* was amplified from DNA of the RH strain of T. gondii, using primers 1 and 2 (Table S3). These primers were designed to remove the predicted signal peptide of 25 aa at the N-terminus and to allow cloning via homologous recombination (SLiCE) (128) into pQE90S.

The *TgLDH1* (TGME49_232350) coding sequence was amplified via PCR using Q5 polymerase (NEB) from cDNA prepared from RH tachyzoites using primers 3 and 4. The PCR fragment was cloned via SLiCE into the linearized pAviTag vector (Lucigen), resulting in a fusion with a C-terminal AviTag followed by a 6×His tag.

For doxycycline-inducible expression of C-terminally 6×His tagged versions of TgLEA8x0, the genes (amplified from the pQE90S-TgLEA8x0 plasmids) were cloned into a ccdB-containing derivative of expression plasmid pASK-IBA1 (IBA, Göttingen, Germany) via Gibson assembly, using primers 5 and 6 for the vector (thereby removing the ompA secretion signal from pASK-IBA1); primers 7 and 8 for TgLEA860, and primers 9 to 11 as forward primers and primer 12 as a shared reverse primer (since the C termini of them are identical) for the other three *TgLEAs*. pASG-IBA33-TgSAG1-His (129) expresses doxycycline-inducible TgSAG1 and was used as the control.

### Expression and purification of recombinant proteins.

BL2(DE3) E. coli bearing the pQE90S-TgLEA8x0-His expression plasmids were grown to an optical density at 600 nm (OD_600_) of ~0.6 to 0.8 in 500 mL LB medium supplemented with 100 μg/mL ampicillin at 250 rpm and 37°C. Expression was induced with 0.5 mM IPTG overnight at 18°C or for 3 h at 37°C (the latter resulting in higher yields, especially for TgLEA860). Bacteria were harvested by centrifugation at 6,000 × *g* for 20 min at 4°C. Pellets were resuspended in 10 mL ice-cold lysis buffer (50 mM sodium phosphate, 300 mM NaCl, 10 mM imidazole, pH 8.0) supplemented with cOmplete protease inhibitor cocktail (Roche), sonicated with a Sonopuls HD70 sonicator and centrifuged at 10,000 × *g* for 10 min at 4°C. The supernatant was clarified by passing through a 0.45-μm syringe filter. Batch purification was performed using either HIS-select nickel affinity gel (Sigma-Aldrich) or 1 mL HisTrap HP columns (GE Healthcare) according to the manufacturer’s instructions. Proteins were eluted with elution buffer (50 mM sodium phosphate, 500 mM NaCl, 250 mM imidazole, pH 8.0). When required, buffer exchange of purified proteins was performed via centrifugal concentrators with PBS using Vivaspin 2 PES columns (Sartorius, Germany) with a molecular weight cutoff (MWCO) of 10,000, according to the manufacturer’s instructions. Purification success was confirmed by SDS-PAGE and subsequent Coomassie staining (see below), and protein concentration was determined using a Qubit fluorometer and the Qubit protein assay kit according to the manufacturer’s instructions. Proteins were aliquoted and stored at –20°C until further use.

Recombinant expression of TgLDH1 in E. coli LOBSTR strain (Kerafast, Boston, USA) was mostly similar to TgLEA purification with minor adjustments. Precultures and expressing cultures were incubated at 15°C in constant rotation at 200 rpm. Expression was induced with rhamnose at a final concentration of 0.2% and allowed to proceed for 24 h at 15°C. Cells were harvested and processed as described above. Purification success was confirmed via SDS-PAGE and subsequent Coomassie staining, resulting in highly pure protein (>10 mg/L of culture) (Fig. 5B, insert).

### Size exclusion chromatography.

Size exclusion chromatography (SEC) of purified TgLEA8x0 was performed on either a Superdex 200 10/300 GL column for TgLEA860 or a Superdex 70 10/300 GL column for the other three proteins using an Äkta purifier FPLC system (GE Healthcare). Calibration of the columns was performed with a calibration kit (GE Healthcare) according to the manufacturer’s instructions, at a flow rate of 0.7 mL/min in PBS at room temperature and within 24 h after protein purification. Chromatograms were analyzed and peak positions determined using UNICORN 5.31 software (GE Healthcare).

### *In vivo* protein stability assay.

To assess the *in vivo* stability of TgLEA860, E. coli strain JW5132-3 bearing the pASK1-TgLEA860-His expression plasmid was grown to an OD_600_ of ~0.5 in 100 mL LB medium supplemented with 100 μg/mL ampicillin at 37°C. Protein expression was induced with 200 ng/mL doxycycline for 1 h before *de novo* protein synthesis was blocked by adding 30 μg/mL chloramphenicol. At the indicated time points, 5 mL samples for SDS-PAGE/Western blot analysis were taken and the OD_600_ was measured. The samples were centrifuged for 5 min at 4,000 × *g*, pellets resuspended in 500 μL urea sample buffer (125 mM Tris/HCl, pH 6.8, 8 M urea, 4% SDS, 0.0025% bromophenol blue) containing 50 mM TCEP. The samples were kept on ice for the length of the experiment and then incubated at 900 rpm for 1 h at room temperature in a thermal shaker. Heating of urea-containing samples was avoided to circumvent the risk of carbamylation. The samples were sonicated for 5 min before loading equal amounts (based on OD_600_) on a 15% polyacrylamide gel (see below).

### Thermal stability assay.

To assess the thermal stability of Tg8x0LEAs, purified proteins, together with TgLDH1, were diluted to 500 μg/mL in PBS, and 40 μL were heated at 95°C for 5 min in a thermal shaker. The insoluble aggregate was separated by centrifugation at 21,500 × *g* for 15 min at 4°C. The supernatant (soluble fraction) was carefully removed, and the pellet (insoluble fraction) was washed twice with PBS. For immunoblotting, 5× Laemmli sample buffer or urea sample buffer was added to the untreated input samples and the supernatants, the pellets were resuspended in 1× Laemmli sample buffer or urea sample buffer, and all samples were heated at 95°C for 5 min prior to loading on a 15% gel.

### LDH aggregation assay.

First, 600 μL mixes of porcine (p)LDH or TgLDH1 (final concentration: 12.5 μM), alone or together with TgLEA, or BSA as control (final concentration 25 μM) were prepared in PBS in 1.5 mL reaction tubes. The samples were allowed to settle for 5 min and 100 μL were transferred to a 96-well quartz plate (Hellma, Germany) for absorbance measurement in an Infinite M200 plate reader (Tecan). This constituted aggregation before freeze-thawing. The samples were then submerged in liquid nitrogen for 30 s and subsequently left at room temperature until completely thawed. Then, 100 μL were transferred to the 96-well quartz plate and the absorbance at 280 nm and 340 nm was recorded. This cycle was repeated three more times. The aggregation index (AI) for each sample after each freeze-thaw cycle was calculated using the following formula: AI = A_340_/(A_280_ – A_340_).

### Differential scanning fluorimetry.

Differential scanning fluorimetry was performed using the GloMelt thermal shift protein stability kit (Biotium, USA). Master mixes containing 9 μL of the GloMelt working solution (prepared according to the manufacturer’s instructions) and TgLEA8x0 at a final concentration of 15 μM, or the IgG control included with the kit at the proposed final concentration of 0.5 μg/mL, were prepared on ice and the volume adjusted to 90 μL with HEPES buffer. Four 20-μL replicates were transferred to a 96-well qPCR plate, covered with corresponding strips and kept on ice. The plate was quickly spun down for 1 min at 200 × *g* and immediately transferred to a CFX96 Touch Real-Time PCR reader (Bio-Rad) for analysis. The following melting curve parameters were applied: preincubation = 2 min at 0°C; range = 0 to 99°C; increments = 0.5°C/min; read interval = 1 min. Data were exported to .txt format and analyzed using the R package ggplot2 0.9.0 and the stat_smooth function.

### Cold stress effect on E. coli viability.

The doxycycline-inducible expression plasmids were transformed into the E. coli strain BW25113 (obtained from the Coli Genetic Stock Center, Yale, USA) (74). Influence of cold stress on E. coli viability was tested using the growth-based viability assay by Qiu et al. (75) in 96-well plate format (Fig. S6). Bacteria were grown to an OD_600_ of ~0.6 to 0.8 in 50 mL LB medium supplemented with 25 μg/mL timentin (as a more stringent replacement for ampicillin [130]) at 37°C (Fig. S6A). Protein expression was induced with 200 ng/mL doxycycline for 1 h at 37°C. Then, 1 mL of induced or uninduced culture was harvested by centrifugation at 4,000 × *g* for 5 min, and pellets were resuspended in PBS to obtain approximately equal cell concentrations (OD_600_ of 0.025). A 3-fold serial dilution with 6 dilution steps was prepared by mixing 100 μL of the bacteria suspension with 200 μL PBS. These samples were the basis for measuring a calibration curve, where the first dilution step was regarded as 100% viability, the second dilution step as 33.33% viability, etc. Then, 50 μL of each dilution were transferred into wells of a 96-well plate. This plate was measured immediately and served as the pre stress/calibration curve measurement. Importantly, calibration curves were experimentally determined for every experimental condition (for each protein and for both uninduced and induced samples). In a second plate, for each sample, 50 μL each of the first dilution step were added in 6 wells (technical replicates). This second plate was then stored in a 4°C cold room for 7 d and subsequently measured. Technical replicates were randomly distributed over the plate, and the R package Well-Plate Maker (WPM) (131) available at the Bioconductor repository (https://bioconductor.org/packages/wpm) was used to design the assay layout. The edge wells were not used but filled with PBS to minimize error from water evaporation. For growth-based viability measurement, 150 μL prewarmed LB medium with 25 μg/mL timentin were added to all wells (doxycycline was omitted for regrowth measurements). To prevent condensation, lids were coated with 3 to 5 mL of 20% EtOH + 0.05% Triton X-100 for 30 s, the solution was poured off and lids were left to dry in laminar flow hood. The covered plate was transferred to a prewarmed microplate reader (Tecan Infinite M200 Pro). The plate was shaken for 550 s with an amplitude of 4 mm at 37°C and the OD_600_ was measured. This was repeated for 100 cycles (ca. 16h).

Data analysis was performed as described by Qiu et al. (75) and using their R script in RStudio (version 1.4.1106). Briefly, the underlying concept of the analysis is that the time required for a bacteria population to reach a certain cell density during exponential growth is related to the number of viable cells at the beginning of bacterial growth (Fig. S6B). An optical density threshold for both plates of one biological replicate and the exponential growth window were determined by algorithms. We used the midpoint thresholding method described by Qiu et al. (75). Then, the time required to reach the threshold cell density was determined for each sample. For simplicity, time was expressed as the cycle number of each reading from the plate reader. The intersection of the growth curve and the threshold was termed fractional cycle number (*C*_t_). To determine the unknown viability of the samples post stress (second plate), the measured *C*_t_ values were fitted to a calibration curve that was taken from a dilution series of the identical samples at day 0 before stress. To construct the calibration curve, the obtained *C*_t_ values (using the same threshold) were plotted against -log_3_ (viability), termed *d*. Linear regression was performed to obtain the calibration curve. The *C*_t_ value of the unknown samples post cold stress was plugged into the calibration curve to determine *d*. Viability was then calculated using 3^−^*^d^*. The viability post stress was plotted as the percentage difference to the uninduced samples. For this purpose, the mean of the viability of the 6 technical replicates was taken and the percentage difference was calculated as 100 × (induced – uninduced)/uninduced.

To analyze growth inhibition before stress, the regrowth of induced samples was also compared to the calibration curves of the uninduced samples. To this end, relative viability of the first dilution step from the induced samples was determined using the calibration curve of the uninduced sample. The percentage difference was calculated as described above. Two-way ANOVA with Sídák multiple-comparison test was performed. The experiment was repeated three times with freshly grown cultures.

### Immunoblotting.

SDS-PAGE and immunoblotting on nitrocellulose membranes were performed using standard techniques. Total protein on blots was visualized using DB71 staining (132) prior to blocking and antibody incubation. Immunostaining was performed using mouse anti-His antibody (MCA1396, Bio-Rad) diluted 1:1,000 in 5% bovine serum albumin in Tris-buffered saline with 0.05% Tween 20 (TBS-T) overnight and antimouse HRP-linked antibody (7076S, Cell Signaling) diluted 1:2,000 in TBS-T for 1 h. HRP signal was detected using ECL Plus Western blotting detection reagents on a Vilber fusion FX imaging system.

### Cell and parasite culture.

All *Toxoplasma* strains were routinely maintained *in vitro* by serial passages in human foreskin fibroblasts (HFFs) as previously described (133).

### Cas9-sgRNA plasmid and repair template construction.

For the generation of a plasmid containing Cas9 and 2 gRNAs against the Late Embryogenesis Abundant cluster (LEAc), the pSAG1::CAS9-U6::sgUPRT plasmid (Addgene plasmid no. 54467) was used, following the protocol described by Long et al. (134) with minor modifications. Briefly, the Uracil phosphoribosyltransferase (UPRT) sgRNA sequence originally present in the pSAG1::CAS9-U6::sgUPRT plasmid was individually replaced with each of the two LEAc sgRNAs (5 and 3) by using the Q5 site-directed mutagenesis kit (NEB, E0554S) with the primer pairs no. 13/14 and 13/15. Subsequently, the portion containing the U6 promoter, sgRNA and gRNA scaffold of one of them (LEAc5) was inserted into the backbone of the second (LEAc3) by restriction enzyme cloning. Namely, the primers 16 and 17 were used to amplify the aforementioned region (676 bp) by PCR. These primers contain the KpnI and XhoI restrictions enzyme sequences, respectively, in opposite orientations at their 5′ end. After PCR amplification and DpnI treatment to remove the vector template, both the LEAc5 U6-gRNA amplicon and the LEAc3 plasmid were cut with KpnI and XhoI restriction enzymes, gel purified, and ligated into the final plasmid (LEAc gRNA3 + 5) containing the Cas9-gRNA scaffold and both LEAc3 and LEAc5 sgRNAs.

The repair template for the LEAc disruption was constructed by using the Q5 hot start high-fidelity DNA assembly kit (New England Biolabs, M0494S) following the manufacturer recommendations and using primers 18 to 23, which contain overhangs complementary to the corresponding adjacent fragment (Table S3). Briefly, 3 fragments consisting in a copy of the hypoxanthine-xanthine-guanine phosphoribosyl transferase gene (*HXGPRT* or *HPT*, TGME49_200320), as well as 5′-end and 3′-end *LEA* homology regions, were amplified by PCR, assembled, and inserted into the universal pUC19 vector that was previously linearized with the restriction enzyme EcoRI. The *HPT* gene coding sequence gene flanked by the 5′ and 3′ UTR regions of the *Toxoplasma* dihydrofolate reductase (*DHFR*) gene was amplified from the pTKO plasmid (135), using the primer pair no. 20/21. Approximately 1 kb of both regions starting at the 5’UTRs of *TgLEA850* and *TgLEA880* (in reverse orientation) were amplified using ME49 gDNA as a template and the primer pairs no. 18/19 and 22/23, respectively. After confirming by Sanger sequencing that the final plasmid did not have any mutation and the assemblage was correct (using primers 24 to 28), the repair template excluding the plasmid backbone (3,995 bp) was amplified by PCR with the primer pair no. 29/30 and purified for transfection in order to increase the efficiency of double homologous recombination (Fig. S6).

### Generation of the *Toxoplasma* ME49 *Δlea*c strain.

To ensure that infections in the cat are successful and yield oocysts, the *Toxoplasma* strain used in our experiments was “cat compatible” (i.e., it was recently obtained from oocysts and maintained *in vitro* for short periods). Moreover, it is of paramount importance to use parasites with low passage numbers for cat infections, as the natural ability of *Toxoplasma* to undergo its sexual cycle in the intestine of a cat has been shown to be compromised when prolonged mouse-to-mouse or *in vitro* passage occurs (136–139). Accordingly, we used an early passage (<15) cat-compatible ME49 strain (ME49 Δ*hpt* luc^+^, kindly provided by Laura Knoll) for knock out. Given that the 4 *TgLEA* genes are located in a gene cluster in the *Toxoplasma* genome on chromosome XII, we used a plasmid containing the CRISPR/Cas9 cassette and 2 gRNAs targeting the 5’ end of the first gene (*TgLEA850*) and the 3’ end of the last gene (*TgLEA880*), and provided a repair template containing a copy of the *HPT* gene flanked by *LEA* homology regions to promote double homologous recombination of the *HPT* gene into the *TgLEA* locus. Thus, approximately 5 × 10^7^ ME49 Δ*hpt* luc tachyzoites were cotransfected with 40 μg of the LEA gRNA5 + 3 plasmid together with 6 μg of a PCR-purified fragment of the pUC-HPT-LEAc repair template to achieve a 5:1 molar ratio. Transfection was performed as previously described (140). After electroporation, parasites were allowed to recover for 15 min and transferred to three different T25 tissue culture flasks containing confluent HFF cells to ensure the obtention of different nonsibling clones. Twenty-four hours after transfection parasites were selected with 25 μg/mL mycophenolic acid (Sigma–Aldrich, cat. no. 89287) and 50 μg/mL xanthine (Sigma–Aldrich, cat. no. X3627) for three lytic cycles and subsequently individual clones were obtained by limiting dilution in 96-well plates. Knockout (KO) clones were verified by PCR for both the absence of internal LEAc amplification and positive bands in the external HPT-LEA PCRs (Fig. S7). All the plasmids and primers used for transfection and for selecting positive clones (F1-3 and R1-3) are listed in Table S3.

### *In vitro* growth assays.

*In vitro* growth assessment of the ME49 Δ*lea*c strain was performed by comparing the invasion rate and plaque area formation with those from the parental ME49 Δ*hpt* strain. Briefly, HFFs were plated in 24-well plates and infected with 200 parasites of each strain. After 7 days, the number of plaques were counted for 4 wells per strain, and the percentage of invading parasites calculated. Furthermore, plaque areas were captured and measured using a Nikon TE2000 inverted microscope equipped with Hamamatsu ORCA-ER digital camera and NIS Elements imaging software, respectively, for at least 25 plaques from 3 different wells for each strain. All experiments were performed in triplicate.

### Animal ethics statement.

All animal experiments were performed in strict accordance with the recommendations in the Guide for the Care and Use of Laboratory Animals of the National Institutes of Health. The Institutional Animal Care and Use Committee (IACUC) at the University of California, Davis (assurance no. A-3433-01) approved all protocols, and all efforts were made to minimize unnecessary distress to the animals. All the kittens used in this study remained healthy throughout each experiment. Because it has been described that *Toxoplasma*-infected cats can reshed oocysts months/years after the initial infection (141, 142), kittens infected with genetically modified *Toxoplasma* strains were euthanized following guidelines from the UC Davis Institutional Biosafety Committee (IBC), National Institutes of Health (NIH), and the Centers for Disease Control and Prevention (CDC), to prevent the potential release of genetically modified *Toxoplasma* strains into the environment.

### Mouse infection.

Four-week-old female CD-1 IGS (Charles River laboratories strain code 022) mice were purchased and used for *in vivo* infections with *Toxoplasma* at 6 weeks of age. Six mice per group (12 mice in total) were injected intraperitoneally with 5,000 freshly harvested tachyzoites of the *Toxoplasma* strains ME49 Δ*hpt* (WT) and ME49 Δ*lea*c (KO). For inoculum preparation, tachyzoites were harvested from *in vitro* culture after 48 to 72 h postinfection (p.i.), when they were still mostly intracellular to avoid excessive loss of viability, and mechanically released from HFFs by successive passage through 27G and 30G needles. Subsequently, parasites were pelleted and resuspended in culture media, and the number of viable tachyzoites manually counted by trypan blue exclusion with a Neubauer chamber. Dilutions were made to achieve the desired number of parasites (5,000) in a 200-μL final volume for injection, and to perform plaque assay to determine parasite viability. Infections were performed within 1 h from harvesting and syringes were kept on ice until injection to reduce viability loss.

Mice were monitored and weighed daily to assess their condition, and those showing signs of severe acute infection (weight loss >20%, inability to reach food or water, severe inactivity/lethargy, or neurological signs such as head tilt, running in circles or ataxia), were euthanized to avoid unnecessary suffering. Mice were sacrificed by CO_2_ asphyxiation followed by cervical dislocation at week 4 p.i. Blood was collected by cardiac puncture for antibody detection, and brains harvested to assess the presence and numbers of cysts (see below).

### Brain cysts detection and counting.

Brains from infected mice were aseptically recovered and homogenized by passage through 18G and 21G needles in 1 mL PBS, and a 100-μL aliquot was taken and incubated for 10 min with 900 μL ice-cold methanol. After washing twice with PBS, brain homogenates were incubated with a 1/500 dilution of Fluorescein-labeled Dolichos biflorus agglutinin (DBA, Vector Laboratories, FL-1031) in PBS for 2 h at 4°C under constant gentle rotation. Brain homogenates were subsequently washed twice and finally resuspended in 1 mL PBS. Next, 100 μL aliquots were taken from each sample and added to individual wells in a 96-well plate to count the number of cysts by fluorescence staining using the FITC channel in a Nikon TE2000 inverted microscope equipped with Hamamatsu ORCA-ER digital camera and NIS Elements imaging software.

### Oocyst production in cats.

Two-month-old specific-pathogen-free DSH kittens (Nutrition and Pet Care Center, Department of Molecular Biosciences, University of California, Davis) were used for oocyst production. Prior to infection, kittens were screened by routine fecal centrifugal floatation to rule out the presence of Toxoplasma gondii oocysts as well as other common feline intestinal parasites, including protozoa such as *Hammondia* spp., *Cystoisospora* spp., *Cryptosporidium* spp., or Giardia spp., which could interfere with *Toxoplasma* experimental infection. No oocysts/cysts were seen in any of the kittens prior to infection. Kittens were infected by feeding them a mixture of brains isolated from infected mice with canned cat food and allowing them to voluntarily eat it. Feces were analyzed daily from day 4 to 10 p.i. by routine fecal floatation, and once oocysts were detected, all feces were collected and processed daily to recover oocysts. Briefly, feces were broken up and thoroughly mixed with 0.1% Tween 80 to form a smooth slurry at approximately 10 mL of Tween per gram of feces. Subsequently, this mixture was filtered by pouring it through a double-layered gauze placed in a fine-mesh tea strainer into 50-mL conical tubes and centrifuged at 1,000 × *g* for 10 min. The supernatant was decanted, and the fecal pellet thoroughly resuspended in a NaCl saturated solution to a final volume of 35 mL. After centrifugation as above, the top 20 to 25 mL supernatant containing floating oocysts was carefully transferred to new tubes and further washed with 0.1% Tween 80 and distilled water. The resultant final pellets containing oocysts were resuspended in a 2% sulfuric acid solution and transferred to T75 tissue culture vented-cap flasks to allow for sporulation at room temperature with aeration and gently rocking for 7 to 10 days. A 10-μL aliquot of the final sulfuric acid oocyst suspension was used to estimate the number of oocysts per cat and day by microscopic examination in a Neubauer chamber. After sporulation, oocysts were counted again, and the rate of sporulation (sporulated oocysts/total number of oocysts) was calculated. Sporulated oocysts were stored at 4°C until use.

All procedures entailing oocysts were conducted in a dedicated biohazard hood and all fecal suspensions were discarded in cat litter containers and subsequently autoclaved. Reusable materials, such as Neubauer chambers, were decontaminated by placing them in a baker with boiling water for 5 min, which is sufficient to inactivate oocysts (28, 110). After oocyst excystation (see below), samples from each of the strains were tested to confirm by PCR that oocysts were indeed from the correct strain and no contamination or mislabeling occurred.

### Resistance treatments and oocyst excystation.

Treatments were conducted on oocysts within 3 months after being shed to ensure they remained as viable as possible, allowing in turn comparable results within experiments. At the time of the assay, for each strain and experimental treatment a similar number of oocysts (~1 to 2 × 10^5^) was added to individual tubes and washed with PBS to remove sulfuric acid. Subsequently, oocysts were resuspended in PBS (for the temperature treatments), in saturated NaCl solution (ρ ~1.20 g/cm^3^ or ~26% wt/vol in distilled water), in 50 to 20% bleach solution (Clorox 1:2 to 1:5 dilution in PBS or ~1.2 to 3% NaOCl) or the pellet was left in the open tube to allow drying. PBS tubes were left either at room temperature (RT) or incubated at 37°C, 40°C, 42°C, or at –20°C for the duration of the treatment. After the incubation time (24 h, 10, 15, or 20 days), tubes were centrifuged to collect the oocysts and perform experiments (see below). For the NaCl condition, the solution was diluted 1:5 by addition of PBS before centrifugation to prevent losses by floatation.

Previous studies have devised different strategies to efficiently excystate *Toxoplasma* oocysts using either mechanical (e.g., grinding, ultrasounds or beads) or chemical (e.g., bleach, bile, taurocholate) means, alone or in combination (110, 137, 143–148). For example, Villegas et al. (110) showed that by using a combination of a physical and chemical treatment with bleach, glass beads and bovine bile, a high percentage of sporozoites are released and able to invade cells. Indeed, early in the excystation process, bleach removes the outer layer of the oocysts, which is only visible ultramicroscopically (18, 23, 47, 143, 149, 150), while glass beads physically break oocysts and release sporocysts. Finally, bile treatment stimulates sporozoites to leave the sporocysts, which break open (143, 146). In our study, we decided to use a similar approach with a few modifications, such as not including the bleach pretreatment, since we did not know whether this may affect LEA knockout oocysts in some way, and decreasing the bile incubation time (see below). The latter modification was done because, in our hands, incubation with 5% ox bile for periods longer than 60 min yielded a considerable decrease in excystation efficiency, as opposed to that described by Villegas et al. (110), where they obtained maximum efficiency after 90 min of incubation.

Therefore, oocysts collected after the resistance treatments were resuspended in 1 mL PBS and added into a 2-mL screw cap tube containing ~300 to 500 mg of 0.5 mm glass beads (Z250465, Sigma-Aldrich). To mechanically rupture the oocysts and free sporocysts, tubes were vortexed 4 times in one-min intervals. The contents of the tubes were carefully transferred to a clean tube and centrifuged at 2,500 × *g* for 6 min. Pellets containing sporocysts and oocysts were resuspended in a 5% ox bile solution (Millipore Sigma, cat no. 70168) and incubated at 37°C for 30 to 45 min. Bile solution was removed by centrifugation at 400 × *g* for 10 min, the pellet containing sporozoites and sporocysts were resuspended in 1 mL medium and immediately inoculated into 6-well plates with confluent HFFs, using 3 to 4 different doses, ranging from the equivalent of 500 to 20,000 starting number of oocysts per well. Parasites were allowed to grow undisturbed for 8 days at 37°C and 5% CO_2_, after which the number of plaques per well were counted. The percentage of excystation was calculated by dividing the number of plaques observed by the theoretical number of excystated oocysts added in the well. An average of this excystation value was calculated for each condition by combining the results obtained for each dose. Subsequently, results for each condition were normalized with the respective untreated (RT) condition and are shown as percentage of theoretical maximum survival.

### Immunofluorescence assay.

Coverslips with HFF monolayers to which oocysts and excystated oocysts were added were incubated for 18 to 24 h and fixed in 4% formaldehyde for 15 min at RT. Subsequently, coverslips were washed with 1× PBS, and blocking/permeabilization was achieved by incubating in PBS containing 0.1% (vol/vol) Triton X-100, 5% (vol/vol) goat serum and 3% (wt/vol) bovine serum albumin (BSA) for 1 h. Coverslips were then incubated for 1 h at RT with a Toxoplasma gondii rabbit polyclonal antibody (Invitrogen PA1-7252) at 1:3,000 dilution after which each well was washed 3 times with PBS, followed by incubation with goat anti rabbit Alexa fluor 488 (1:3,000 dilution, Invitrogen) and Hoechst 33258 (1:3,000 dilution) for 1 h. Finally, coverslips were washed 3 times with PBS, mounted with VectaShield antifade mounting medium (Vector Laboratories ref. H-1000) and observed under an inverted fluorescence microscope (Eclipse Ti-S; Nikon) equipped with a digital camera (CoolSNAP EZ; Roper Scientific), and NIS Elements imaging software. It is important to note that since the PA1-7252 commercial antibody is polyclonal and no stage-specific markers are indicated, it is likely that both sporozoites and tachyzoites can be labeled.

### Statistical analyses.

All statistical analyses were performed using Prism (GraphPad) versions 8.0 or 9.0. Data are presented as mean ± standard deviation (SD) unless otherwise noted, and in all cases, *P* < 0.05 was considered significant. To compare the parental and KO strains in the *in vitro* growth assays and oocyst resistance trials, an unpaired *t* test was used followed by Welch's correction for each condition, and a two-way ANOVA with Sídák multiple-comparison test was performed for the bacterial growth assay. R package ggplot2 0.9.0 and the stat_smooth function was used to analyze the differential scanning fluorimetry data, showing 95% CI of SEM.
