# Supplementary figures and images for: Late Embryogenesis Abundant Proteins Contribute to the Resistance of Toxoplasma gondii Oocysts against Environmental Stresses
Source: mBio. 2023 Feb 21;14(2):e02868-22. doi: 10.1128/mbio.02868-22 (PMC10128015; doi:10.1128/mbio.02868-22)

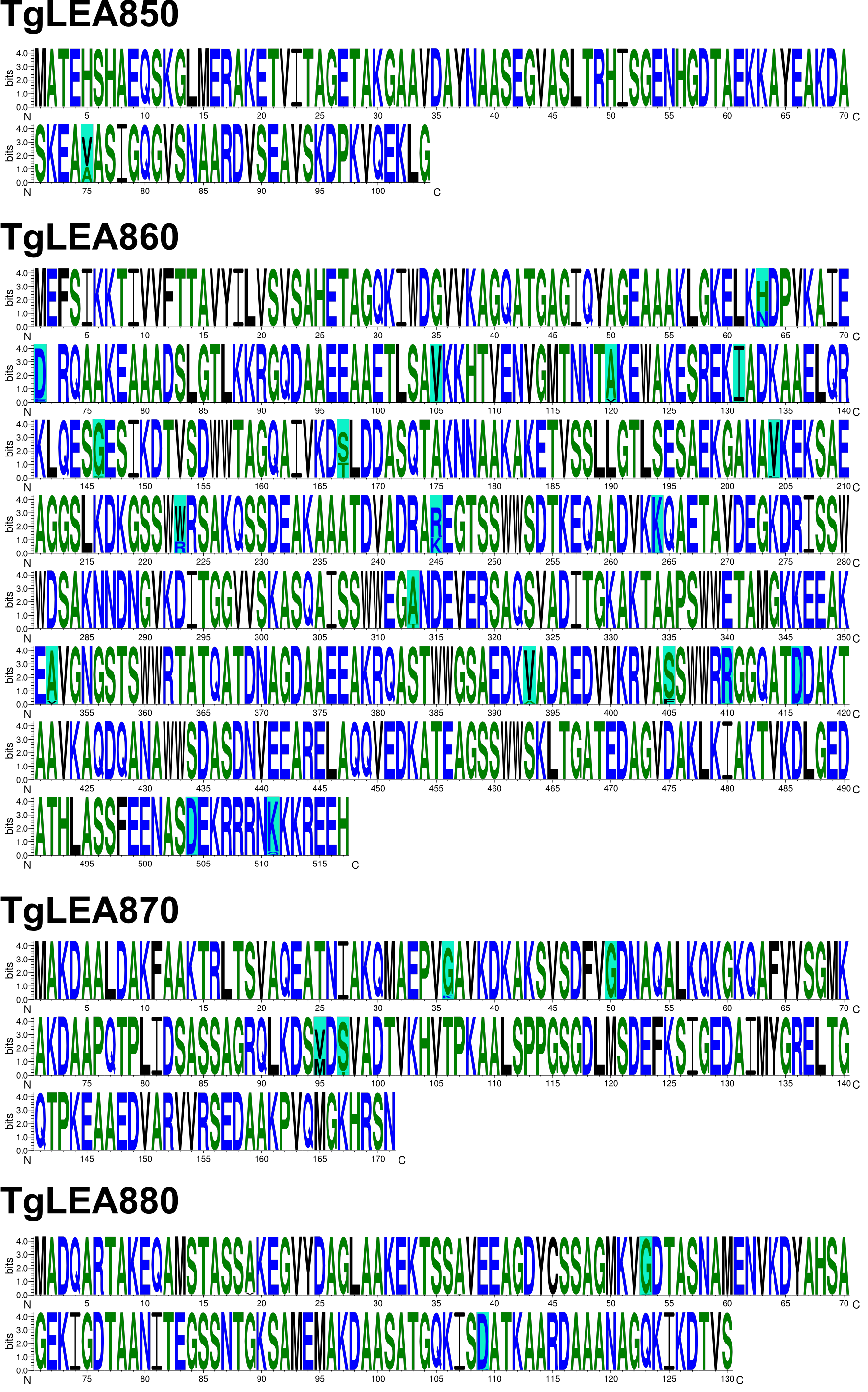

Supplement: FIG S1 [file mbio.02868-22-s0004.tif]

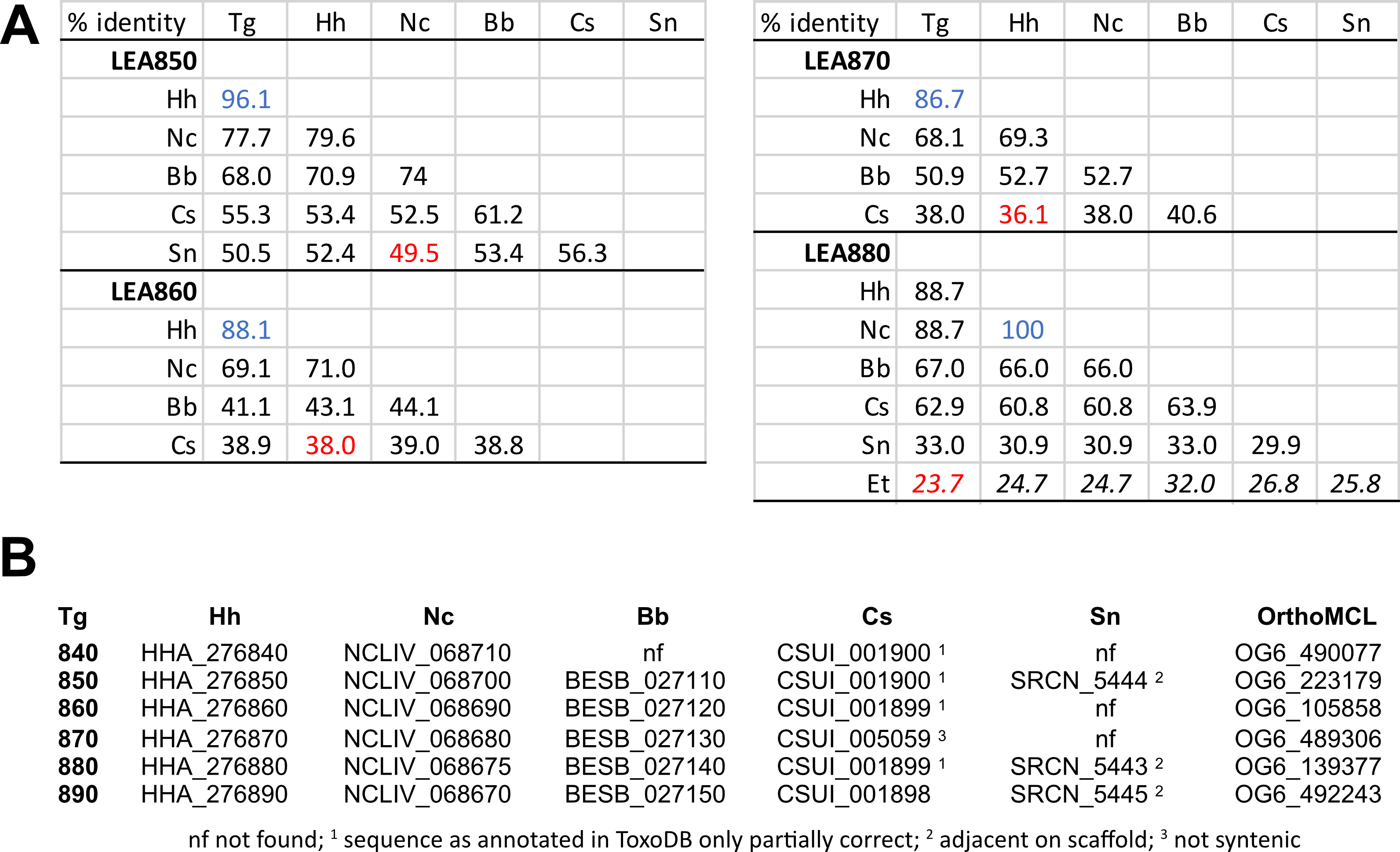

Supplement: FIG S2 [file mbio.02868-22-s0005.tif]

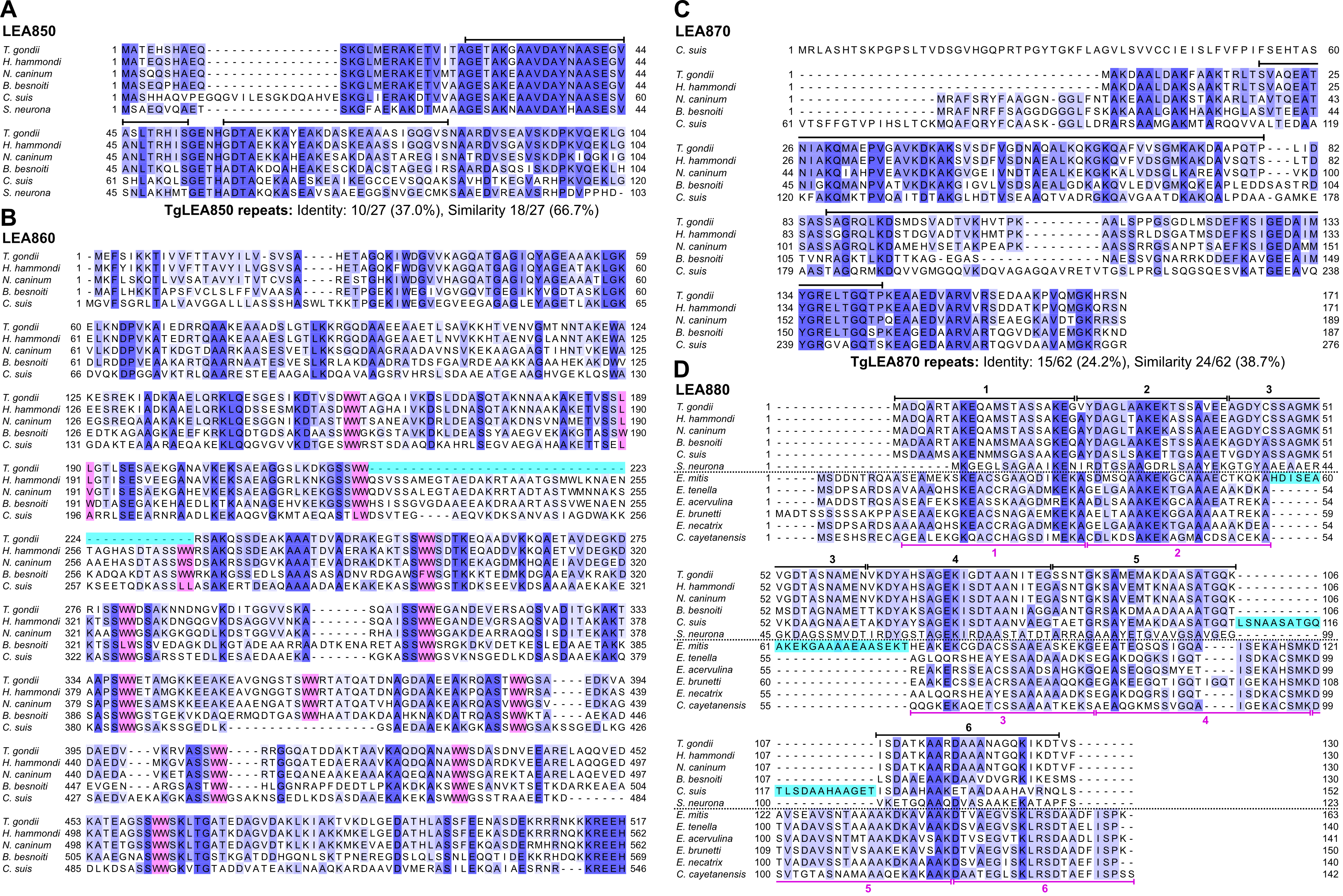

Supplement: FIG S3 [file mbio.02868-22-s0006.tif]

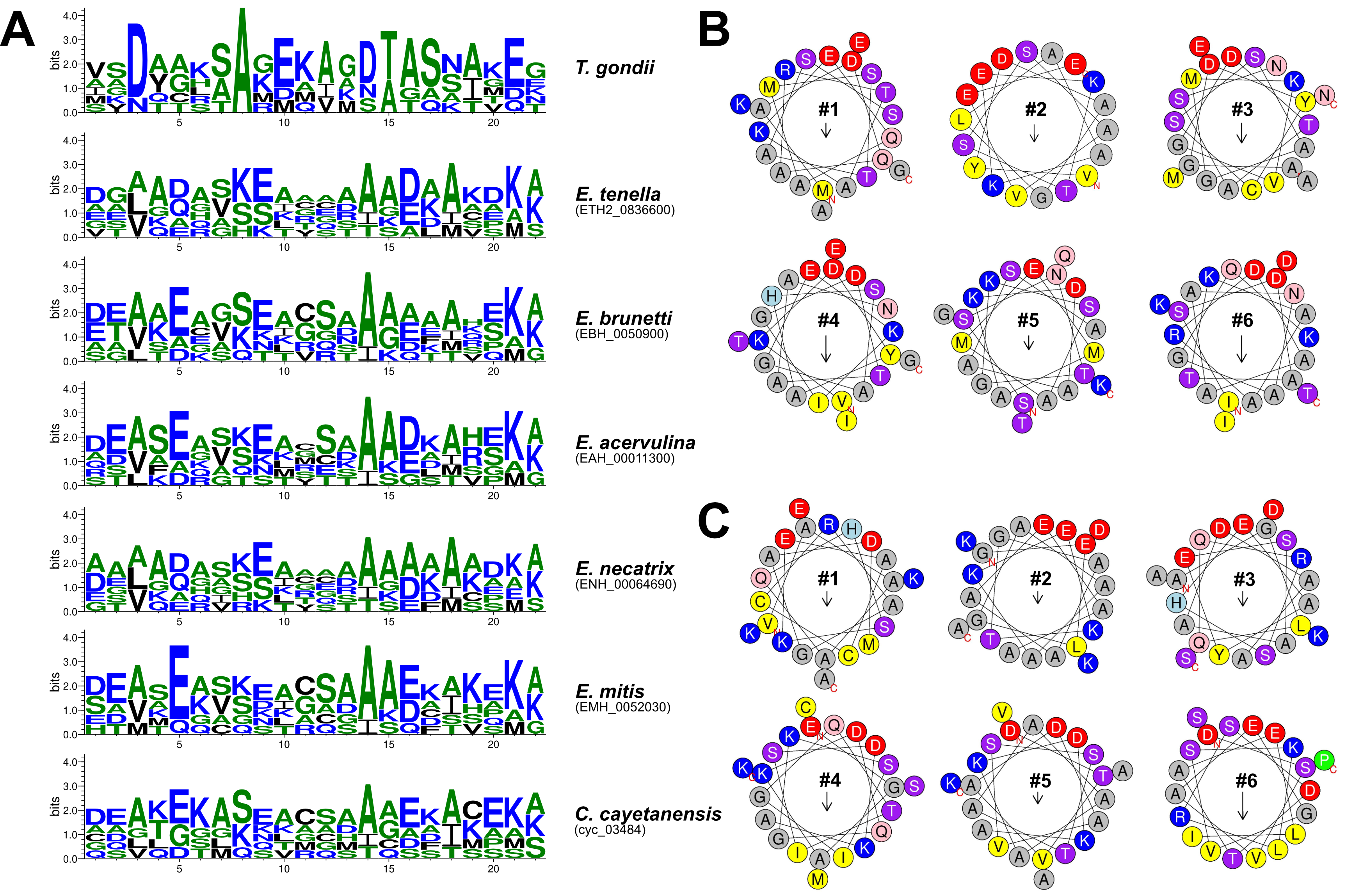

Supplement: FIG S4 [file mbio.02868-22-s0007.tif]

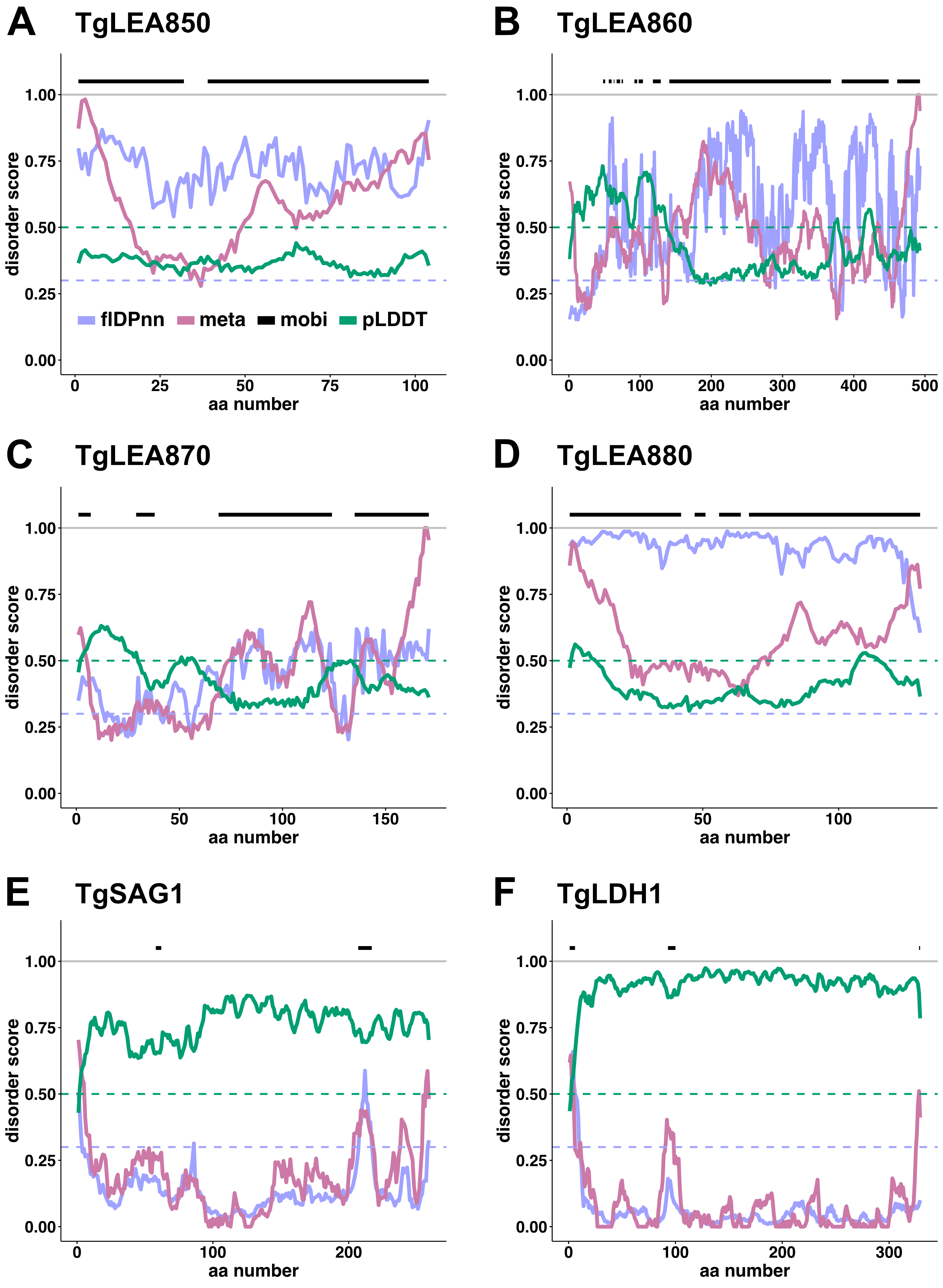

Supplement: FIG S5 [file mbio.02868-22-s0008.tif]

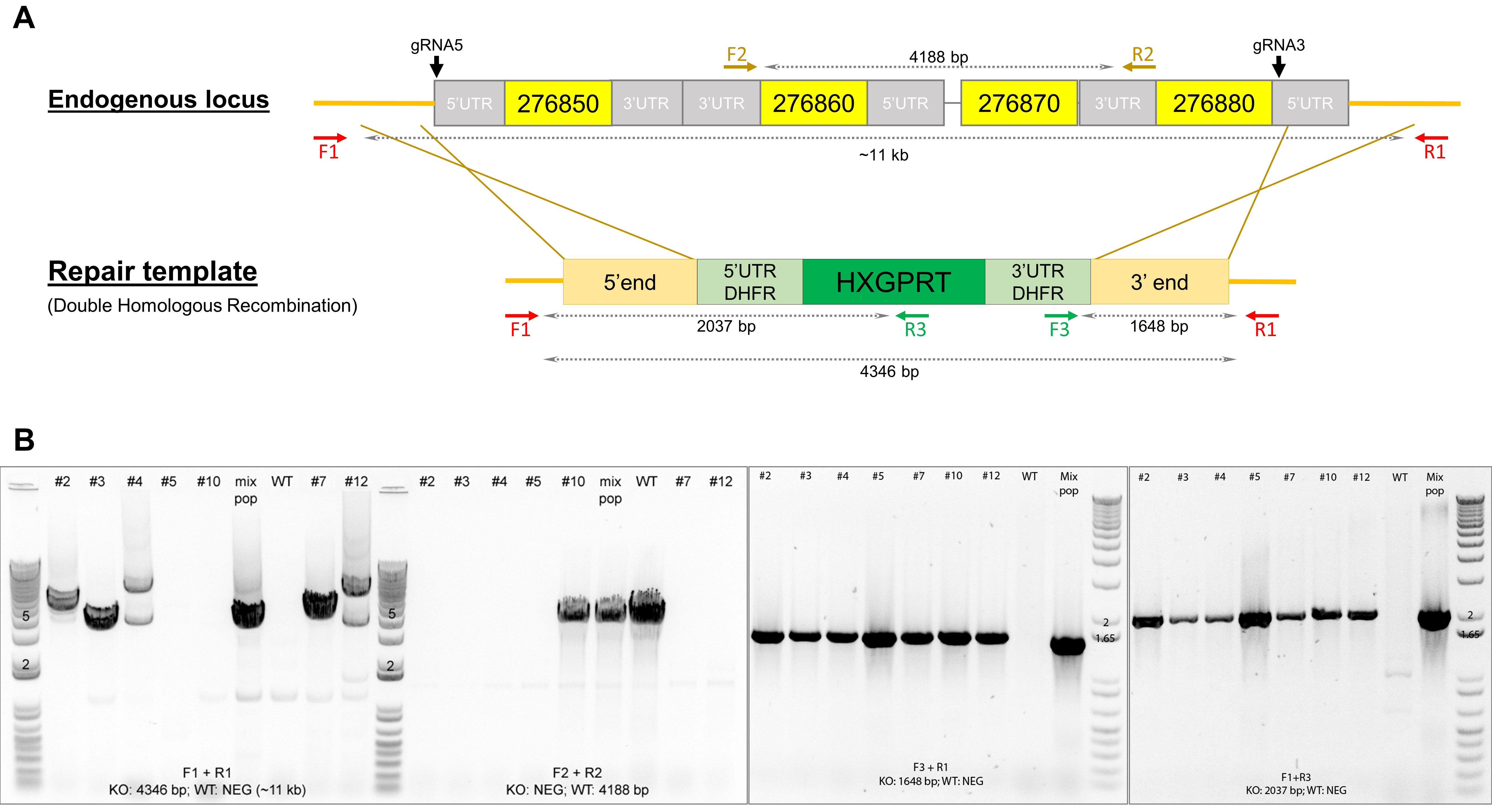

Supplement: FIG S7 [file mbio.02868-22-s0010.tif]
