# Supplementary material for: Late Embryogenesis Abundant Proteins Contribute to the Resistance of Toxoplasma gondii Oocysts against Environmental Stresses
Source: mBio. 2023 Feb 21;14(2):e02868-22. doi: 10.1128/mbio.02868-22 (PMC10128015; doi:10.1128/mbio.02868-22)
Supplement: TABLE S1 [file mbio.02868-22-s0001.docx]

## Table S1. Bioinformatic and biochemical data of TgLEA8x0

|  | TgLEA850 | TgLEA860 | TgLEA870 | TgLEA880 | TgSAG1 | TgLDH1 | Tg all proteins |
| --- | --- | --- | --- | --- | --- | --- | --- |
| ToxoDB ID | [TGME49_276850](http://toxodb.org/toxo/app/record/gene/TGME49_276850) | [TGME49_276860](http://toxodb.org/toxo/app/record/gene/TGME49_276860) | [TGME49_276870](http://toxodb.org/toxo/app/record/gene/TGME49_276870) | [TGME49_276880](http://toxodb.org/toxo/app/record/gene/TGME49_276880) | TGME49_233460 | TGME49_232350 |  |
| LEAP DB class ^1^ | - | 6 ^9^ | - | - | n.a. | n.a. |  |
| MobiDbB disorder % ^2^ | 54.8 | 25.1 | 33.3 | 60.0 | 0 | 0 |  |
| Das-Pappu phase ^3^ | 2 | 2 | 2 | 2 | 1 | 1 |  |
| Size (aa) | 104 | 517 | 171 | 130 | 336 | 329 |  |
| Theoretical MW (kDa) | 10.71 | 52.9 (-sp) | 17.92 | 13.05 | 26.9 (-sp) | 35.5 |  |
| Observed MW by SEC (kDa) | 41 | 90 (152) | 73 (43) | 100 (56/26) | n.d. | n.d. |  |
| Signal peptide ^4^ | no | yes | no | no | yes | no (but NLS) |  |
| Isoelectric point ^5^ | 5.67 | 5.29 | 9.39 | 4.96 | 5.64 | 6.06 |  |
| GRAVY-KD ^6^ | -0.67 | -0.82 | -0.48 | -0.60 | -0.18 | 0.14 | -0.42 |
| GRAVY-IDP ^6^ | 0.46 | 0.79 | 0.5 | 0.44 | 1.29 | 1.62 | 1.04 |
| PFAM ^6^ | - | - | - | - | n.d. | n.d. |  |
| Interpro ^6^ | - | IPR004238 | - | - | n.d. | n.d. |  |
| PantherDB ^6^ | - | PTHR47372 | - | - | n.d. | n.d. |  |
| RiboProf Median combined ^7^ | 0 | 0 | 0 | 0 | 3750.64 | 671.55 |  |
| mean essentiality score ^8^ | 0.26 | -0.95 | 0.59 | 0.61 | -0.07 | 0.78 |  |
| aa in % for W/Y/F^9^ | 0/1.92/0 | 4.64/0.39/0.58 | 0/0.58/2.34 | 0/2.31/0 | 0.89/0.89/3.57 | 0.3/3.65/2.43 | 1.01/1.46/3.48 |

1 Hunault, G. & Jaspard, E. LEAPdb: a database for the late embryogenesis abundant proteins. *BMC Genomics* **11**, 221, doi:10.1186/1471-2164-11-221 (2010).

2 Piovesan, D. *et al.* MobiDB: intrinsically disordered proteins in 2021. *Nucleic Acids Res* **49**, D361-d367, doi:10.1093/nar/gkaa1058 (2021).

3 Holehouse, A. S., Das, R. K., Ahad, J. N., Richardson, M. O. G. & Pappu, R. V. CIDER: Resources to Analyze Sequence-Ensemble Relationships of Intrinsically Disordered Proteins. *Biophysj* **112**, 16-21, doi:10.1016/j.bpj.2016.11.3200 (2017).

4 Almagro Armenteros, J. J. *et al.* SignalP 5.0 improves signal peptide predictions using deep neural networks. *Nat Biotechnol* **37**, 420-423, doi:10.1038/s41587-019-0036-z (2019).

5 Kozlowski, L. P. IPC - Isoelectric Point Calculator. *Biol Direct* **11**, 55, doi:10.1186/s13062-016-0159-9 (2016).

6 see Methods for details

7 Hassan, M. A., Vasquez, J. J., Guo-Liang, C., Meissner, M. & Siegel, T. N. Comparative ribosome profiling uncovers a dominant role for translational control in Toxoplasma gondii. *BMC Genomics* **18**, 961, doi:10.1186/s12864-017-4362-6 (2017).

8 Sidik, S. M. *et al.* A genome-wide CRISPR screen in Toxoplasma identifies essential apicomplexan genes. *Cell* **166**, 1423-1435.e1412, doi:10.1016/j.cell.2016.08.019 (2016).

9 aa 129-176: EKIADKAAELQRKLQESGESIKDTVSDWWTAGQAIVKDSLDDASQTAK

generic class 6 motif (Perl regular expressions; see ToxoDB): [^LNP][^G][ADEGILMQRSTVY][AEKQRSTY].[KR][AT].[ADENT][^DP][EGIKLMQST].{1,67}[^DER][^AS]K[AD][^IL][^N].[^E]?.{1,6}G?
