# Supplementary material for: Late Embryogenesis Abundant Proteins Contribute to the Resistance of Toxoplasma gondii Oocysts against Environmental Stresses
Source: mBio. 2023 Feb 21;14(2):e02868-22. doi: 10.1128/mbio.02868-22 (PMC10128015; doi:10.1128/mbio.02868-22)
Supplement: TABLE S2 [file mbio.02868-22-s0002.docx]

## Table S2. Predicted consensus phosphorylation sites

| aa | TgLEA850^1^ | TgLEA860^1^ | TgLEA870^1^ | TgLEA880^1^ |
| --- | --- | --- | --- | --- |
| **Ser** | 6, 52, 71, 77, 90, 94 | 56, 100, 121, 161, 167, 169, 181, 187, 193, 194, 198, 202, 203, 222, 251, 252, 356, 378, 469, 470, 476 | 85, 86, 94, 97, 112, 121, 126, 156 | 14, 17, 18, 35, 36, 91, 101 |
| **Thr** | 24 | 59, 147, 228, 301, 388 | 78, 101, 106, 142 | 7, 103, 111, 128 |
| **Tyr** | 37 | - | 134 | 24, 44, 66 |

^1^ residue numbers potentially phosphorylated
