# Supplementary material for: Late Embryogenesis Abundant Proteins Contribute to the Resistance of Toxoplasma gondii Oocysts against Environmental Stresses
Source: mBio. 2023 Feb 21;14(2):e02868-22. doi: 10.1128/mbio.02868-22 (PMC10128015; doi:10.1128/mbio.02868-22)
Supplement: FIG S6 [file mbio.02868-22-s0009.pdf]

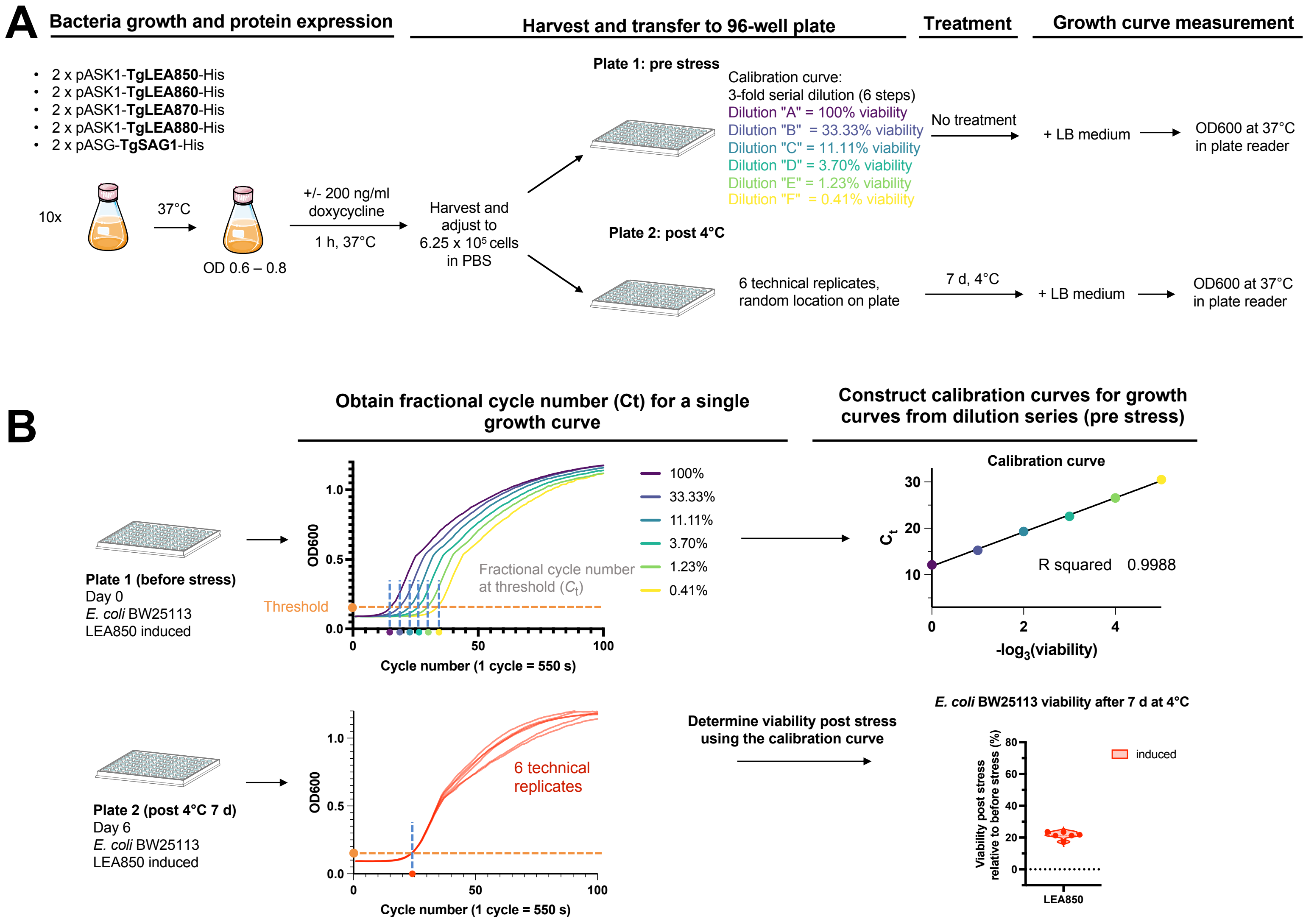

A
Bacteria growth and protein expression
Harvest and transfer to 96-well plate
Treatment
Growth curve measurement
2 x pASK1-TgLEA850-His
2 x pASK1-TgLEA860-His
2 x pASK1-TgLEA870-His
2 x pASK1-TgLEA880-His
2 x pASG-TgSAG1-His
10x
37°C
OD 0.6 – 0.8
+/- 200 ng/ml doxycycline
1 h, 37°C
Harvest and adjust to 6.25 x 10^5 cells in PBS
Plate 1: pre stress
Calibration curve:
3-fold serial dilution (6 steps)
Dilution "A" = 100% viability
Dilution "B" = 33.33% viability
Dilution "C" = 11.11% viability
Dilution "D" = 3.70% viability
Dilution "E" = 1.23% viability
Dilution "F" = 0.41% viability
No treatment
+ LB medium
OD600 at 37°C in plate reader
Plate 2: post 4°C
6 technical replicates, random location on plate
7 d, 4°C
+ LB medium
OD600 at 37°C in plate reader
B
Obtain fractional cycle number (Ct) for a single growth curve
Construct calibration curves for growth curves from dilution series (pre stress)
Plate 1 (before stress)
Day 0
E. coli BW25113
LEA850 induced
Threshold
OD600
1.0
0.5
0.0
0
50
100
Cycle number (1 cycle = 550 s)
Fractional cycle number at threshold (Ct)
100%
33.33%
11.11%
3.70%
1.23%
0.41%
Calibration curve
Ct
30
20
10
0
2
4
R squared 0.9988
-log3(viability)
Plate 2 (post 4°C 7 d)
Day 6
E. coli BW25113
LEA850 induced
OD600
1.0
0.5
0.0
0
50
100
Cycle number (1 cycle = 550 s)
6 technical replicates
Determine viability post stress using the calibration curve
E. coli BW25113 viability after 7 d at 4°C
Viability post stress relative to before stress (%)
80
60
40
20
0
induced
LEA850

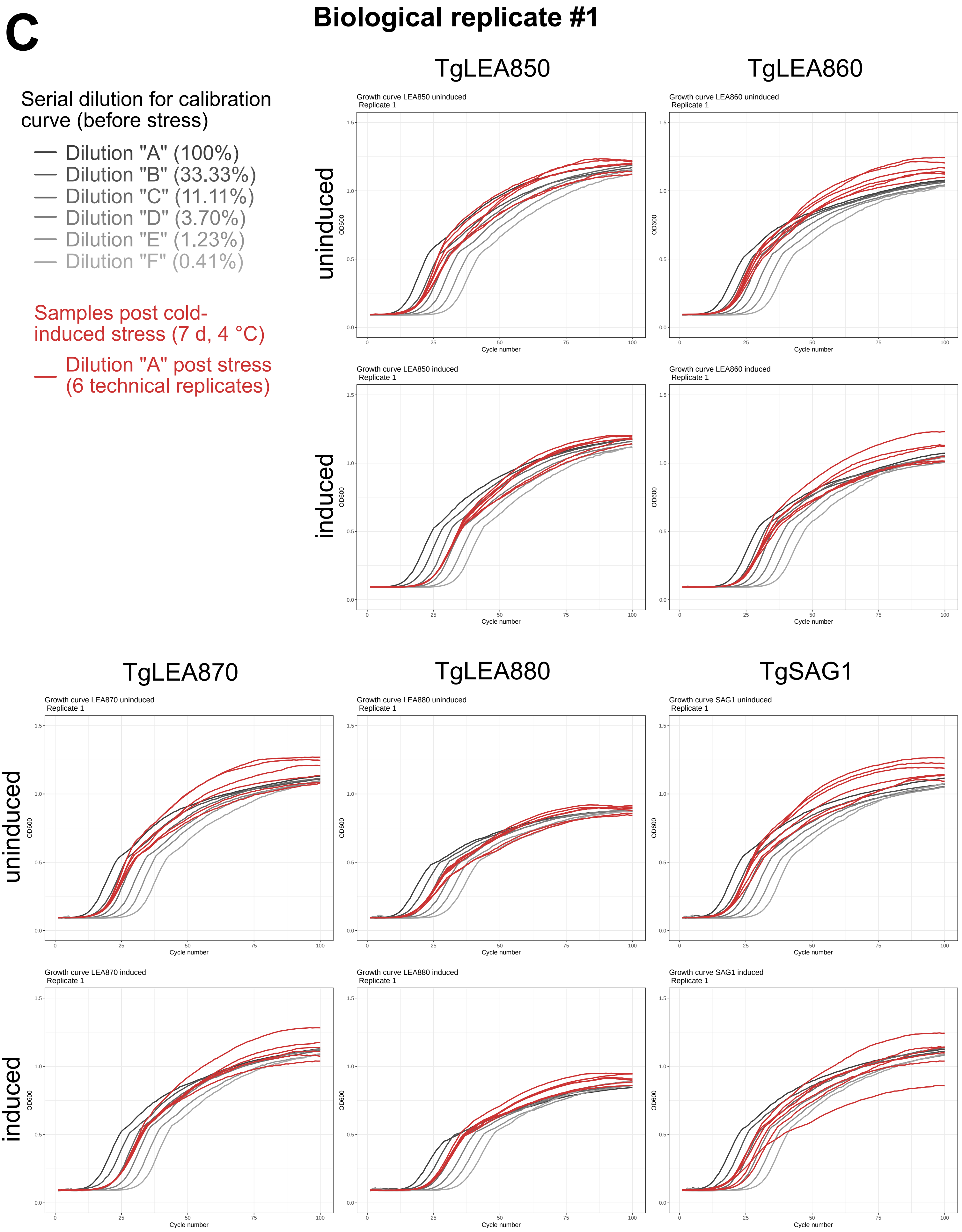
C
Biological replicate #1
Serial dilution for calibration curve (before stress)
Dilution "A" (100%)
Dilution "B" (33.33%)
Dilution "C" (11.11%)
Dilution "D" (3.70%)
Dilution "E" (1.23%)
Dilution "F" (0.41%)
Samples post cold-induced stress (7 d, 4 °C)
Dilution "A" post stress (6 technical replicates)
TgLEA850
TgLEA860
TgLEA870
TgLEA880
TgSAG1
uninduced
induced
Growth curve LEA850 uninduced Replicate 1
Growth curve LEA860 uninduced Replicate 1
Growth curve LEA850 induced Replicate 1
Growth curve LEA860 induced Replicate 1
Growth curve LEA870 uninduced Replicate 1
Growth curve LEA880 uninduced Replicate 1
Growth curve SAG1 uninduced Replicate 1
Growth curve LEA870 induced Replicate 1
Growth curve LEA880 induced Replicate 1
Growth curve SAG1 induced Replicate 1
OD600
Cycle number
0.0
0.5
1.0
1.5
0
25
50
75
100

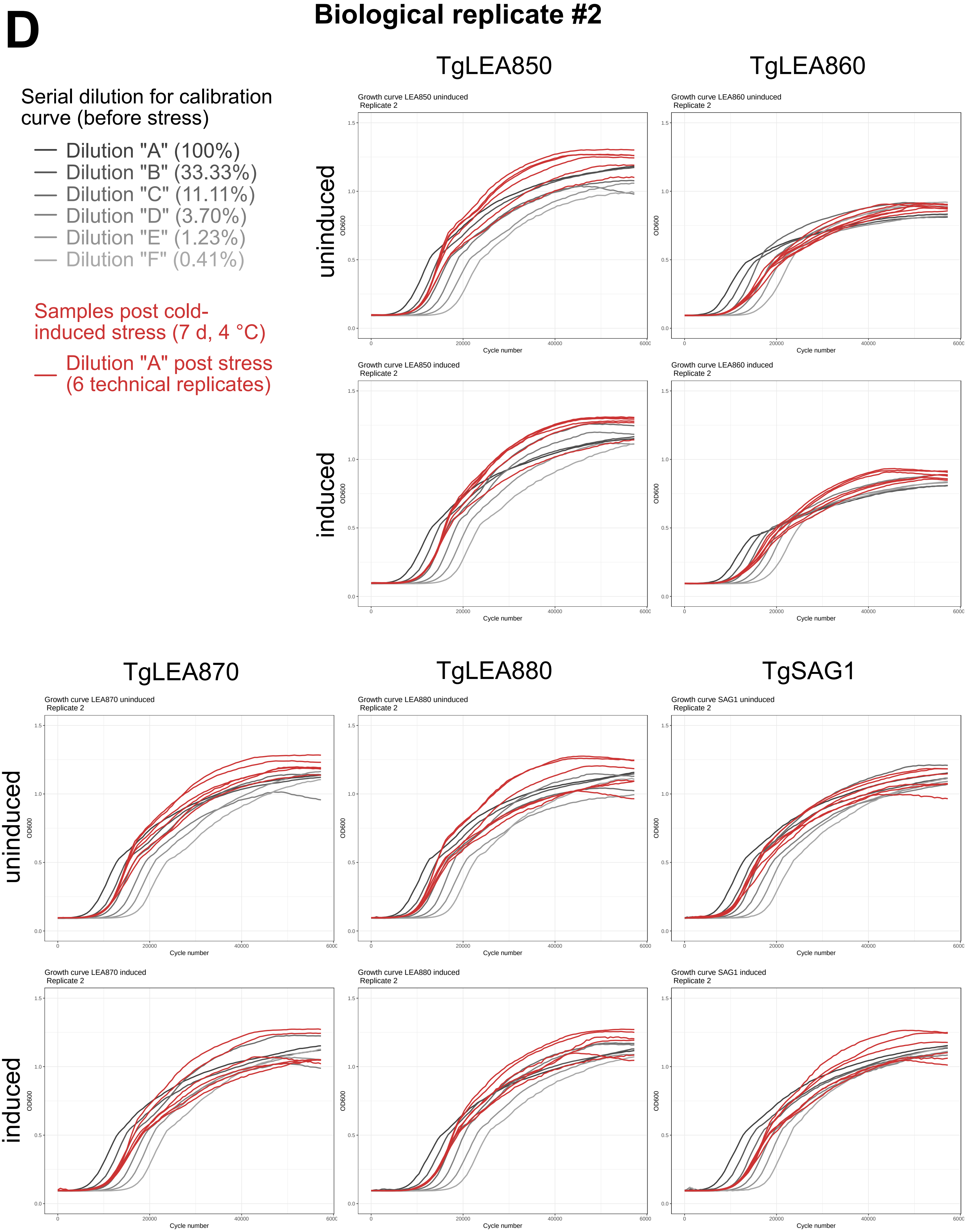
D
Biological replicate #2
Serial dilution for calibration curve (before stress)
Dilution "A" (100%)
Dilution "B" (33.33%)
Dilution "C" (11.11%)
Dilution "D" (3.70%)
Dilution "E" (1.23%)
Dilution "F" (0.41%)
Samples post cold-induced stress (7 d, 4 °C)
Dilution "A" post stress (6 technical replicates)
TgLEA850
TgLEA860
TgLEA870
TgLEA880
TgSAG1
uninduced
induced
Growth curve LEA850 uninduced Replicate 2
Growth curve LEA860 uninduced Replicate 2
Growth curve LEA850 induced Replicate 2
Growth curve LEA860 induced Replicate 2
Growth curve LEA870 uninduced Replicate 2
Growth curve LEA880 uninduced Replicate 2
Growth curve SAG1 uninduced Replicate 2
Growth curve LEA870 induced Replicate 2
Growth curve LEA880 induced Replicate 2
Growth curve SAG1 induced Replicate 2
OD600
Cycle number
0.0
0.5
1.0
1.5
0
20000
40000

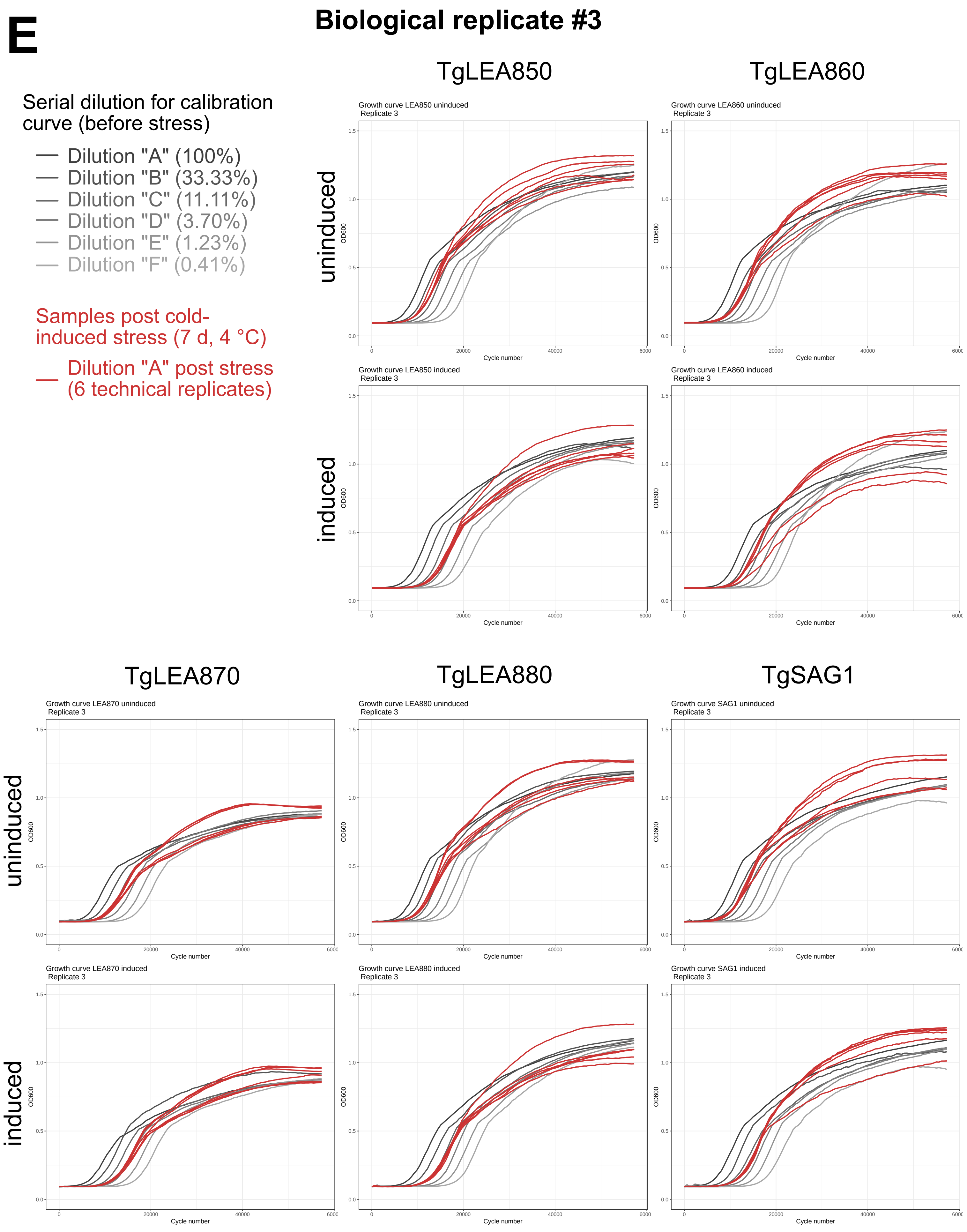
E
Biological replicate #3
Serial dilution for calibration curve (before stress)
Dilution "A" (100%)
Dilution "B" (33.33%)
Dilution "C" (11.11%)
Dilution "D" (3.70%)
Dilution "E" (1.23%)
Dilution "F" (0.41%)
Samples post cold-induced stress (7 d, 4 °C)
Dilution "A" post stress (6 technical replicates)
TgLEA850
TgLEA860
uninduced
induced
Growth curve LEA850 uninduced Replicate 3
Growth curve LEA860 uninduced Replicate 3
Growth curve LEA850 induced Replicate 3
Growth curve LEA860 induced Replicate 3
TgLEA870
TgLEA880
TgSAG1
Growth curve LEA870 uninduced Replicate 3
Growth curve LEA880 uninduced Replicate 3
Growth curve SAG1 uninduced Replicate 3
Growth curve LEA870 induced Replicate 3
Growth curve LEA880 induced Replicate 3
Growth curve SAG1 induced Replicate 3
OD600
Cycle number
0.0
0.5
1.0
1.5
0
20000
40000
