# Supplementary material for: Late Embryogenesis Abundant Proteins Contribute to the Resistance of Toxoplasma gondii Oocysts against Environmental Stresses
Source: mBio. 2023 Feb 21;14(2):e02868-22. doi: 10.1128/mbio.02868-22 (PMC10128015; doi:10.1128/mbio.02868-22)
Supplement: TABLE S3 [file mbio.02868-22-s0003.docx]

## Table S3: Primers used in the present study

| Primer Name | Primer ID | Sequence (5' to 3')^1,2^ | Gene/Plasmid target | Purpose |
| --- | --- | --- | --- | --- |
| LEA_860_SLiC-fwd | Primer #1 | ATTCATTAAAGAGGAGAAATTACATATGGAAACAGCCGGACAAAA | TgLEA860 (in CDs) | Cloning into pQE90S vector |
| LEA_860_SLiC-rev | Primer #2 | TTAAGCATTCTGCCGACATGGAAGCTTAATGGTGATGGTGATGGTG CTCCTCCCGTTTTTTTT | TgLEA860 (3'UTR) at the stop codon | Cloning into pQE90S vector |
| LDH-1_SL-fwd | Primer #3 | AAGAAGGAGATATACATATGGCACCCGCACTTGTGCAGAG | TgLDH1 (TGME49_232350) | Cloning into pAviTag vector |
| LDH-1_SL-rev | Primer #4 | GTCGGCGGGGTGGATAAGCTCGCCTGAAGAGCAGCAACCG | TgLDH1 (TGME49_232350) | Cloning into pAviTag vector |
| pASK1-fwd | Primer #5 | TAATAAGCTTGACCTGTGAAGTGAA | pASK-IBA1 | Gibson assembly |
| pASK1-rev | Primer #6 | TTTTTGCCCTCGTTATCTAGATTTT | pASK-IBA1 | Gibson assembly |
| pASK-860-fwd | Primer #7 | AAAATCTAGATAACGAGGGCAAAAAATGGAAACAGCCGGACAAAA | pQE90S-TgLEA860 | Gibson assembly |
| pASK-860-rev | Primer #8 | TTCACTTCACAGGTCAAGCTTATTAATGGTGATGGTGATGGTGCT | pQE90S-TgLEA860 | Gibson assembly |
| pASK-850-fwd | Primer #9 | AAAATCTAGATAACGAGGGCAAAAAATGGCAACTGAGCACTCTCA | pQE90S-TgLEA850 | Gibson assembly |
| pASK-870-fwd | Primer #10 | AAAATCTAGATAACGAGGGCAAAAAATGGCAAAAGATGCAGCACT | pQE90S-TgLEA870 | Gibson assembly |
| pASK-880-fwd | Primer #11 | AAAATCTAGATAACGAGGGCAAAAAATGGCAGATCAGGCACGTAC | pQE90S-TgLEA880 | Gibson assembly |
| pASK-858788-rev | Primer #12 | TTCACTTCACAGGTCAAGCTTATTAGTGATGGTGATGGTGATGCA | pQE90S-TgLEA850/70/80 | Gibson assembly |
| Q5gRNA-Rv | Primer #13 | AACTTGACATCCCCATTTAC | pSAG1-Cas9-sgUPRT | Q5 mutagenesis |
| Q5gRNALEA5-Fw | Primer #14 | GTTCGGACTGAAAGCCACGCGTTTTAGAGCTAGAAATAGC | pSAG1-Cas9-sgUPRT | Q5 mutagenesis |
| Q5gRNALEA3-Fw | Primer #15 | GCGAATGGTTGGTAAACCTAGTTTTAGAGCTAGAAATAGC | pSAG1-Cas9-sgUPRT | Q5 mutagenesis |
| gRNA2-KpnI-Fw | Primer #16 | AATTG*GGTACC*CAAGTAAGCAGAAGCACGCTG | pSAG1-Cas9-sgUPRT | Subcloning to have 2 gRNAs in a single plasmid. |
| gRNA2-XhoI-Rv | Primer #17 | TCGAC*CTCGAG*AATTAACCCTCACTAAAGGg | pSAG1-Cas9-sgUPRT | Subcloning to have 2 gRNAs in a single plasmid. |
| LEAc5GibpUC-Fw | Primer #18 | cgacgttgtaaaacgacggccagtgGTCTGAGGTCGGTCTCTTGC | gDNA (LEAc5 end, 3UTR 276860. Overhang is pUC19) | Gibson assembly |
| LEAc5GibHPT-Rv | Primer #19 | aggtttcgtgctgAAGACTGGGTTTTCGGTGTG | gDNA (LEAc5 end, 3UTR 276860. Overhang is 5'UTR DHFR) | Gibson assembly |
| HPTGibLEAc5-Fw | Primer #20 | aaaacccagtcttCAGCACGAAACCTTGCATTC | pTKO (HPT repair cassette, 5'UTR DHFR. Overhang is LEAc5 end, 3UTR 276860) | Gibson assembly |
| HPTGibLEAc3-Rv | Primer #21 | ctgacgtagcgttGTGTCACTGTAGCCTGCCAG | pTKO (HPT repair cassette, 3'UTR DHFR. Overhang is LEAc3 end, 5UTR 276880) | Gibson assembly |
| LEAc3GibHPT-Fw | Primer #22 | gctacagtgacacAACGCTACGTCAGCAAGTGATAATAC | gDNA (LEAc3 end, 5UTR 276880. Overhang is 3'UTR DHFR) | Gibson assembly |
| LEAc3GibpUC-Rv | Primer #23 | agaggatccccgggtaccgagctcgATAGCGACACAGGCCAATTC | gDNA (LEAc3 end, 5UTR 276880. Overhang is pUC19) | Gibson assembly |
| 3'UTRDHFR-Rv | Primer #24 | CACTGTAGCCTGCCAGAACA | 3'UTR DHFR from pTKO plasmid | Sequencing/PCR |
| 2768805'UTR-(Rv) | Primer #25 | TGAACGTCCAGTCGTTGGGG | TGME49_276880 (5'UTR) | Sequencing/PCR |
| LEAc5-Fw | Primer #26 | CACACCGAAAACCCAGTCTT | LEA cluster 5' end (TGME49_276860, 3'UTR ) | Sequencing/PCR |
| LEAc3-Rv | Primer #27 | GTAGCCTCTGGCAAAACAGC | LEA cluster 3' end (TGME49_276880, 5'UTR) | Sequencing/PCR |
| HPT-Fw | Primer #28 | CTTACTTCGGCGAGGAGTTG | HXGPRT CDs | Sequencing/PCR |
| LEAc5HOM-Fw | Primer #29 | GTCTGAGGTCGGTCTCTTGC | LEA cluster 5' end (TGME49_276860, 3'UTR ) | PCR of repair template |
| LEAc3HOM-Rv | Primer #30 | ATAGCGACACAGGCCAATTC | LEA cluster 3' end (TGME49_276880, 5'UTR) | PCR of repair template |
| 2768603'UTR-Rv | Primer F1 | CTCAAACAGCACGTGACAGC | TGME49_276860 (3'UTR) | Diagnostic PCR |
| 2768905'UTR-Rv | Primer R1 | TGTGCAATTAAACGGCAGAA | TGME49_276890 (5'UTR) | Diagnostic PCR |
| 2768603'UTR-Rv2 | Primer F2 | AGTGGTAACTGCCAGGATGG | TGME49_276860 (3'UTR) | Diagnostic PCR |
| 2768803'UTR-Fw | Primer R2 | ATTTCTTCTTCGCTGCGTGT | TGME49_276880 (3'UTR) | Diagnostic PCR |
| 3'UTRDHFR-Fw | Primer F3 | ACGATGTGCTGTCAGTTTCG | 3'UTR DHFR from pTKO plasmid | Diagnostic PCR |
| HPT-Rv | Primer R3 | TGTCGCGAAAGATTGACAAG | HXGPRT CDs | Diagnostic PCR |

^1^Lower case indicates overhangs for Gibson assembly efficiency

^2^restriction enzyme sequences are indicated in italics.
